# Supplementary material for: Does (mis)communication mitigate the upshot of diversity?
Source: PLoS One. 2023 Mar 24;18(3):e0283248. doi: 10.1371/journal.pone.0283248 (PMC10038257; doi:10.1371/journal.pone.0283248)
Supplement: S1 File — (DOCX) [file pone.0283248.s001.docx]

**Manhattan Distance Measure**

MH20Manrug4relaya <- HPDFinal$'relay.randoms'[HPDFinal$SmoothingFactor=="4" & HPDFinal$'max.heur.number'=="20"]

MH20Manrug4relayb <- HPDFinal$'relay.experts'[HPDFinal$SmoothingFactor=="4" & HPDFinal$'max.heur.number'=="20"]

t.test(MH20Manrug4relaya, MH20Manrug4relayb, paired=T)

MH20Manrug6relaya <- HPDFinal$'relay.randoms'[HPDFinal$SmoothingFactor=="6" & HPDFinal$'max.heur.number'=="20"]

MH20Manrug6relayb <- HPDFinal$'relay.experts'[HPDFinal$SmoothingFactor=="6" & HPDFinal$'max.heur.number'=="20"]

t.test(MH20Manrug6relaya, MH20Manrug6relayb, paired=T)

MH20Manrug8relaya <- HPDFinal$'relay.randoms'[HPDFinal$SmoothingFactor=="8" & HPDFinal$'max.heur.number'=="20"]

MH20Manrug8relayb <- HPDFinal$'relay.experts'[HPDFinal$SmoothingFactor=="8" & HPDFinal$'max.heur.number'=="20"]

t.test(MH20Manrug8relaya, MH20Manrug8relayb, paired=T)

MH20Manrug10relaya <- HPDFinal$'relay.randoms'[HPDFinal$SmoothingFactor=="10" & HPDFinal$'max.heur.number'=="20"]

MH20Manrug10relayb <- HPDFinal$'relay.experts'[HPDFinal$SmoothingFactor=="10" & HPDFinal$'max.heur.number'=="20"]

t.test(MH20Manrug10relaya, MH20Manrug10relayb, paired=T)

MH20Manrug12relaya <- HPDFinal$'relay.randoms'[HPDFinal$SmoothingFactor=="12" & HPDFinal$'max.heur.number'=="20"]

MH20Manrug12relayb <- HPDFinal$'relay.experts'[HPDFinal$SmoothingFactor=="12" & HPDFinal$'max.heur.number'=="20"]

t.test(MH20Manrug12relaya, MH20Manrug12relayb, paired=T)

MH20Manrug14relaya <- HPDFinal$'relay.randoms'[HPDFinal$SmoothingFactor=="14" & HPDFinal$'max.heur.number'=="20"]

MH20Manrug14relayb <- HPDFinal$'relay.experts'[HPDFinal$SmoothingFactor=="14" & HPDFinal$'max.heur.number'=="20"]

t.test(MH20Manrug14relaya, MH20Manrug14relayb, paired=T)

MH20Manrug16relaya <- HPDFinal$'relay.randoms'[HPDFinal$SmoothingFactor=="16" & HPDFinal$'max.heur.number'=="20"]

MH20Manrug16relayb <- HPDFinal$'relay.experts'[HPDFinal$SmoothingFactor=="16" & HPDFinal$'max.heur.number'=="20"]

t.test(MH20Manrug16relaya, MH20Manrug16relayb, paired=T)

MH20Manrug4tourna <- HPDFinal$'tourn.randoms'[HPDFinal$SmoothingFactor=="4" & HPDFinal$'max.heur.number'=="20"]

MH20Manrug4tournb <- HPDFinal$'tourn.experts'[HPDFinal$SmoothingFactor=="4" & HPDFinal$'max.heur.number'=="20"]

t.test(MH20Manrug4tourna, MH20Manrug4tournb, paired=T)

MH20Manrug6tourna <- HPDFinal$'tourn.randoms'[HPDFinal$SmoothingFactor=="6" & HPDFinal$'max.heur.number'=="20"]

MH20Manrug6tournb <- HPDFinal$'tourn.experts'[HPDFinal$SmoothingFactor=="6" & HPDFinal$'max.heur.number'=="20"]

t.test(MH20Manrug6tourna, MH20Manrug6tournb, paired=T)

MH20Manrug8tourna <- HPDFinal$'tourn.randoms'[HPDFinal$SmoothingFactor=="8" & HPDFinal$'max.heur.number'=="20"]

MH20Manrug8tournb <- HPDFinal$'tourn.experts'[HPDFinal$SmoothingFactor=="8" & HPDFinal$'max.heur.number'=="20"]

t.test(MH20Manrug8tourna, MH20Manrug8tournb, paired=T)

MH20Manrug10tourna <- HPDFinal$'tourn.randoms'[HPDFinal$SmoothingFactor=="10" & HPDFinal$'max.heur.number'=="20"]

MH20Manrug10tournb <- HPDFinal$'tourn.experts'[HPDFinal$SmoothingFactor=="10" & HPDFinal$'max.heur.number'=="20"]

t.test(MH20Manrug10tourna, MH20Manrug10tournb, paired=T)

MH20Manrug12tourna <- HPDFinal$'tourn.randoms'[HPDFinal$SmoothingFactor=="12" & HPDFinal$'max.heur.number'=="20"]

MH20Manrug12tournb <- HPDFinal$'tourn.experts'[HPDFinal$SmoothingFactor=="12" & HPDFinal$'max.heur.number'=="20"]

t.test(MH20Manrug12tourna, MH20Manrug12tournb, paired=T)

MH20Manrug14tourna <- HPDFinal$'tourn.randoms'[HPDFinal$SmoothingFactor=="14" & HPDFinal$'max.heur.number'=="20"]

MH20Manrug14tournb <- HPDFinal$'tourn.experts'[HPDFinal$SmoothingFactor=="14" & HPDFinal$'max.heur.number'=="20"]

t.test(MH20Manrug14tourna, MH20Manrug14tournb, paired=T)

MH20Manrug16tourna <- HPDFinal$'tourn.randoms'[HPDFinal$SmoothingFactor=="16" & HPDFinal$'max.heur.number'=="20"]

MH20Manrug16tournb <- HPDFinal$'tourn.experts'[HPDFinal$SmoothingFactor=="16" & HPDFinal$'max.heur.number'=="20"]

t.test(MH20Manrug16tourna, MH20Manrug16tournb, paired=T)

MH20Manrug4Normtourna <- HPDFinal$'tourn.randoms.miscommunication'[HPDFinal$SmoothingFactor=="4" & HPDFinal$'max.heur.number'=="20" & HPDFinal$'distance.type'=="manhattan" & HPDFinal$'distribution'=="normal"]

MH20Manrug4Normtournb <- HPDFinal$'tourn.experts.miscommunication'[HPDFinal$SmoothingFactor=="4" & HPDFinal$'max.heur.number'=="20" & HPDFinal$'distance.type'=="manhattan" & HPDFinal$'distribution'=="normal"]

t.test(MH20Manrug4Normtourna, MH20Manrug4Normtournb, paired=T)

MH20Manrug6Normtourna <- HPDFinal$'tourn.randoms.miscommunication'[HPDFinal$SmoothingFactor=="6" & HPDFinal$'max.heur.number'=="20" & HPDFinal$'distance.type'=="manhattan" & HPDFinal$'distribution'=="normal"]

MH20Manrug6Normtournb <- HPDFinal$'tourn.experts.miscommunication'[HPDFinal$SmoothingFactor=="6" & HPDFinal$'max.heur.number'=="20" & HPDFinal$'distance.type'=="manhattan" & HPDFinal$'distribution'=="normal"]

t.test(MH20Manrug6Normtourna, MH20Manrug6Normtournb, paired=T)

MH20Manrug8Normtourna <- HPDFinal$'tourn.randoms.miscommunication'[HPDFinal$SmoothingFactor=="8" & HPDFinal$'max.heur.number'=="20" & HPDFinal$'distance.type'=="manhattan" & HPDFinal$'distribution'=="normal"]

MH20Manrug8Normtournb <- HPDFinal$'tourn.experts.miscommunication'[HPDFinal$SmoothingFactor=="8" & HPDFinal$'max.heur.number'=="20" & HPDFinal$'distance.type'=="manhattan" & HPDFinal$'distribution'=="normal"]

t.test(MH20Manrug8Normtourna, MH20Manrug8Normtournb, paired=T)

MH20Manrug10Normtourna <- HPDFinal$'tourn.randoms.miscommunication'[HPDFinal$SmoothingFactor=="10" & HPDFinal$'max.heur.number'=="20" & HPDFinal$'distance.type'=="manhattan" & HPDFinal$'distribution'=="normal"]

MH20Manrug10Normtournb <- HPDFinal$'tourn.experts.miscommunication'[HPDFinal$SmoothingFactor=="10" & HPDFinal$'max.heur.number'=="20" & HPDFinal$'distance.type'=="manhattan" & HPDFinal$'distribution'=="normal"]

t.test(MH20Manrug10Normtourna, MH20Manrug10Normtournb, paired=T)

MH20Manrug12Normtourna <- HPDFinal$'tourn.randoms.miscommunication'[HPDFinal$SmoothingFactor=="12" & HPDFinal$'max.heur.number'=="20" & HPDFinal$'distance.type'=="manhattan" & HPDFinal$'distribution'=="normal"]

MH20Manrug12Normtournb <- HPDFinal$'tourn.experts.miscommunication'[HPDFinal$SmoothingFactor=="12" & HPDFinal$'max.heur.number'=="20" & HPDFinal$'distance.type'=="manhattan" & HPDFinal$'distribution'=="normal"]

t.test(MH20Manrug12Normtourna, MH20Manrug12Normtournb, paired=T)

MH20Manrug14Normtourna <- HPDFinal$'tourn.randoms.miscommunication'[HPDFinal$SmoothingFactor=="14" & HPDFinal$'max.heur.number'=="20" & HPDFinal$'distance.type'=="manhattan" & HPDFinal$'distribution'=="normal"]

MH20Manrug14Normtournb <- HPDFinal$'tourn.experts.miscommunication'[HPDFinal$SmoothingFactor=="14" & HPDFinal$'max.heur.number'=="20" & HPDFinal$'distance.type'=="manhattan" & HPDFinal$'distribution'=="normal"]

t.test(MH20Manrug14Normtourna, MH20Manrug14Normtournb, paired=T)

MH20Manrug16Normtourna <- HPDFinal$'tourn.randoms.miscommunication'[HPDFinal$SmoothingFactor=="16" & HPDFinal$'max.heur.number'=="20" & HPDFinal$'distance.type'=="manhattan" & HPDFinal$'distribution'=="normal"]

MH20Manrug16Normtournb <- HPDFinal$'tourn.experts.miscommunication'[HPDFinal$SmoothingFactor=="16" & HPDFinal$'max.heur.number'=="20" & HPDFinal$'distance.type'=="manhattan" & HPDFinal$'distribution'=="normal"]

t.test(MH20Manrug16Normtourna, MH20Manrug16Normtournb, paired=T)

MH20Manrug4Normrelaya <- HPDFinal$'relay.randoms.miscommunication'[HPDFinal$SmoothingFactor=="4" & HPDFinal$'max.heur.number'=="20" & HPDFinal$'distance.type'=="manhattan" & HPDFinal$'distribution'=="normal"]

MH20Manrug4Normrelayb <- HPDFinal$'relay.experts.miscommunication'[HPDFinal$SmoothingFactor=="4" & HPDFinal$'max.heur.number'=="20" & HPDFinal$'distance.type'=="manhattan" & HPDFinal$'distribution'=="normal"]

t.test(MH20Manrug4Normrelaya, MH20Manrug4Normrelayb, paired=T)

MH20Manrug6Normrelaya <- HPDFinal$'relay.randoms.miscommunication'[HPDFinal$SmoothingFactor=="6" & HPDFinal$'max.heur.number'=="20" & HPDFinal$'distance.type'=="manhattan" & HPDFinal$'distribution'=="normal"]

MH20Manrug6Normrelayb <- HPDFinal$'relay.experts.miscommunication'[HPDFinal$SmoothingFactor=="6" & HPDFinal$'max.heur.number'=="20" & HPDFinal$'distance.type'=="manhattan" & HPDFinal$'distribution'=="normal"]

t.test(MH20Manrug6Normrelaya, MH20Manrug6Normrelayb, paired=T)

MH20Manrug8Normrelaya <- HPDFinal$'relay.randoms.miscommunication'[HPDFinal$SmoothingFactor=="8" & HPDFinal$'max.heur.number'=="20" & HPDFinal$'distance.type'=="manhattan" & HPDFinal$'distribution'=="normal"]

MH20Manrug8Normrelayb <- HPDFinal$'relay.experts.miscommunication'[HPDFinal$SmoothingFactor=="8" & HPDFinal$'max.heur.number'=="20" & HPDFinal$'distance.type'=="manhattan" & HPDFinal$'distribution'=="normal"]

t.test(MH20Manrug8Normrelaya, MH20Manrug8Normrelayb, paired=T)

MH20Manrug10Normrelaya <- HPDFinal$'relay.randoms.miscommunication'[HPDFinal$SmoothingFactor=="10" & HPDFinal$'max.heur.number'=="20" & HPDFinal$'distance.type'=="manhattan" & HPDFinal$'distribution'=="normal"]

MH20Manrug10Normrelayb <- HPDFinal$'relay.experts.miscommunication'[HPDFinal$SmoothingFactor=="10" & HPDFinal$'max.heur.number'=="20" & HPDFinal$'distance.type'=="manhattan" & HPDFinal$'distribution'=="normal"]

t.test(MH20Manrug10Normrelaya, MH20Manrug10Normrelayb, paired=T)

MH20Manrug12Normrelaya <- HPDFinal$'relay.randoms.miscommunication'[HPDFinal$SmoothingFactor=="12" & HPDFinal$'max.heur.number'=="20" & HPDFinal$'distance.type'=="manhattan" & HPDFinal$'distribution'=="normal"]

MH20Manrug12Normrelayb <- HPDFinal$'relay.experts.miscommunication'[HPDFinal$SmoothingFactor=="12" & HPDFinal$'max.heur.number'=="20" & HPDFinal$'distance.type'=="manhattan" & HPDFinal$'distribution'=="normal"]

t.test(MH20Manrug12Normrelaya, MH20Manrug12Normrelayb, paired=T)

MH20Manrug14Normrelaya <- HPDFinal$'relay.randoms.miscommunication'[HPDFinal$SmoothingFactor=="14" & HPDFinal$'max.heur.number'=="20" & HPDFinal$'distance.type'=="manhattan" & HPDFinal$'distribution'=="normal"]

MH20Manrug14Normrelayb <- HPDFinal$'relay.experts.miscommunication'[HPDFinal$SmoothingFactor=="14" & HPDFinal$'max.heur.number'=="20" & HPDFinal$'distance.type'=="manhattan" & HPDFinal$'distribution'=="normal"]

t.test(MH20Manrug14Normrelaya, MH20Manrug14Normrelayb, paired=T)

MH20Manrug16Normrelaya <- HPDFinal$'relay.randoms.miscommunication'[HPDFinal$SmoothingFactor=="16" & HPDFinal$'max.heur.number'=="20" & HPDFinal$'distance.type'=="manhattan" & HPDFinal$'distribution'=="normal"]

MH20Manrug16Normrelayb <- HPDFinal$'relay.experts.miscommunication'[HPDFinal$SmoothingFactor=="16" & HPDFinal$'max.heur.number'=="20" & HPDFinal$'distance.type'=="manhattan" & HPDFinal$'distribution'=="normal"]

t.test(MH20Manrug16Normrelaya, MH20Manrug16Normrelayb, paired=T)

MH20Manrug4Poisrelaya <- HPDFinal$'relay.randoms.miscommunication'[HPDFinal$SmoothingFactor=="4" & HPDFinal$'max.heur.number'=="20" & HPDFinal$'distance.type'=="manhattan" & HPDFinal$'distribution'=="poisson"]

MH20Manrug4Poisrelayb <- HPDFinal$'relay.experts.miscommunication'[HPDFinal$SmoothingFactor=="4" & HPDFinal$'max.heur.number'=="20" & HPDFinal$'distance.type'=="manhattan" & HPDFinal$'distribution'=="poisson"]

t.test(MH20Manrug4Poisrelaya, MH20Manrug4Poisrelayb, paired=T)

MH20Manrug6Poisrelaya <- HPDFinal$'relay.randoms.miscommunication'[HPDFinal$SmoothingFactor=="6" & HPDFinal$'max.heur.number'=="20" & HPDFinal$'distance.type'=="manhattan" & HPDFinal$'distribution'=="poisson"]

MH20Manrug6Poisrelayb <- HPDFinal$'relay.experts.miscommunication'[HPDFinal$SmoothingFactor=="6" & HPDFinal$'max.heur.number'=="20" & HPDFinal$'distance.type'=="manhattan" & HPDFinal$'distribution'=="poisson"]

t.test(MH20Manrug6Poisrelaya, MH20Manrug6Poisrelayb, paired=T)

MH20Manrug8Poisrelaya <- HPDFinal$'relay.randoms.miscommunication'[HPDFinal$SmoothingFactor=="8" & HPDFinal$'max.heur.number'=="20" & HPDFinal$'distance.type'=="manhattan" & HPDFinal$'distribution'=="poisson"]

MH20Manrug8Poisrelayb <- HPDFinal$'relay.experts.miscommunication'[HPDFinal$SmoothingFactor=="8" & HPDFinal$'max.heur.number'=="20" & HPDFinal$'distance.type'=="manhattan" & HPDFinal$'distribution'=="poisson"]

t.test(MH20Manrug8Poisrelaya, MH20Manrug8Poisrelayb, paired=T)

MH20Manrug10Poisrelaya <- HPDFinal$'relay.randoms.miscommunication'[HPDFinal$SmoothingFactor=="10" & HPDFinal$'max.heur.number'=="20" & HPDFinal$'distance.type'=="manhattan" & HPDFinal$'distribution'=="poisson"]

MH20Manrug10Poisrelayb <- HPDFinal$'relay.experts.miscommunication'[HPDFinal$SmoothingFactor=="10" & HPDFinal$'max.heur.number'=="20" & HPDFinal$'distance.type'=="manhattan" & HPDFinal$'distribution'=="poisson"]

t.test(MH20Manrug10Poisrelaya, MH20Manrug10Poisrelayb, paired=T)

MH20Manrug12Poisrelaya <- HPDFinal$'relay.randoms.miscommunication'[HPDFinal$SmoothingFactor=="12" & HPDFinal$'max.heur.number'=="20" & HPDFinal$'distance.type'=="manhattan" & HPDFinal$'distribution'=="poisson"]

MH20Manrug12Poisrelayb <- HPDFinal$'relay.experts.miscommunication'[HPDFinal$SmoothingFactor=="12" & HPDFinal$'max.heur.number'=="20" & HPDFinal$'distance.type'=="manhattan" & HPDFinal$'distribution'=="poisson"]

t.test(MH20Manrug12Poisrelaya, MH20Manrug12Poisrelayb, paired=T)

MH20Manrug14Poisrelaya <- HPDFinal$'relay.randoms.miscommunication'[HPDFinal$SmoothingFactor=="14" & HPDFinal$'max.heur.number'=="20" & HPDFinal$'distance.type'=="manhattan" & HPDFinal$'distribution'=="poisson"]

MH20Manrug14Poisrelayb <- HPDFinal$'relay.experts.miscommunication'[HPDFinal$SmoothingFactor=="14" & HPDFinal$'max.heur.number'=="20" & HPDFinal$'distance.type'=="manhattan" & HPDFinal$'distribution'=="poisson"]

t.test(MH20Manrug14Poisrelaya, MH20Manrug14Poisrelayb, paired=T)

MH20Manrug16Poisrelaya <- HPDFinal$'relay.randoms.miscommunication'[HPDFinal$SmoothingFactor=="16" & HPDFinal$'max.heur.number'=="20" & HPDFinal$'distance.type'=="manhattan" & HPDFinal$'distribution'=="poisson"]

MH20Manrug16Poisrelayb <- HPDFinal$'relay.experts.miscommunication'[HPDFinal$SmoothingFactor=="16" & HPDFinal$'max.heur.number'=="20" & HPDFinal$'distance.type'=="manhattan" & HPDFinal$'distribution'=="poisson"]

t.test(MH20Manrug16Poisrelaya, MH20Manrug16Poisrelayb, paired=T)

MH20Manrug4Poistourna <- HPDFinal$'tourn.randoms.miscommunication'[HPDFinal$SmoothingFactor=="4" & HPDFinal$'max.heur.number'=="20" & HPDFinal$'distance.type'=="manhattan" & HPDFinal$'distribution'=="poisson"]

MH20Manrug4Poistournb <- HPDFinal$'tourn.experts.miscommunication'[HPDFinal$SmoothingFactor=="4" & HPDFinal$'max.heur.number'=="20" & HPDFinal$'distance.type'=="manhattan" & HPDFinal$'distribution'=="poisson"]

t.test(MH20Manrug4Poistourna, MH20Manrug4Poistournb, paired=T)

MH20Manrug6Poistourna <- HPDFinal$'tourn.randoms.miscommunication'[HPDFinal$SmoothingFactor=="6" & HPDFinal$'max.heur.number'=="20" & HPDFinal$'distance.type'=="manhattan" & HPDFinal$'distribution'=="poisson"]

MH20Manrug6Poistournb <- HPDFinal$'tourn.experts.miscommunication'[HPDFinal$SmoothingFactor=="6" & HPDFinal$'max.heur.number'=="20" & HPDFinal$'distance.type'=="manhattan" & HPDFinal$'distribution'=="poisson"]

t.test(MH20Manrug6Poistourna, MH20Manrug6Poistournb, paired=T)

MH20Manrug8Poistourna <- HPDFinal$'tourn.randoms.miscommunication'[HPDFinal$SmoothingFactor=="8" & HPDFinal$'max.heur.number'=="20" & HPDFinal$'distance.type'=="manhattan" & HPDFinal$'distribution'=="poisson"]

MH20Manrug8Poistournb <- HPDFinal$'tourn.experts.miscommunication'[HPDFinal$SmoothingFactor=="8" & HPDFinal$'max.heur.number'=="20" & HPDFinal$'distance.type'=="manhattan" & HPDFinal$'distribution'=="poisson"]

t.test(MH20Manrug8Poistourna, MH20Manrug8Poistournb, paired=T)

MH20Manrug10Poistourna <- HPDFinal$'tourn.randoms.miscommunication'[HPDFinal$SmoothingFactor=="10" & HPDFinal$'max.heur.number'=="20" & HPDFinal$'distance.type'=="manhattan" & HPDFinal$'distribution'=="poisson"]

MH20Manrug10Poistournb <- HPDFinal$'tourn.experts.miscommunication'[HPDFinal$SmoothingFactor=="10" & HPDFinal$'max.heur.number'=="20" & HPDFinal$'distance.type'=="manhattan" & HPDFinal$'distribution'=="poisson"]

t.test(MH20Manrug10Poistourna, MH20Manrug10Poistournb, paired=T)

MH20Manrug12Poistourna <- HPDFinal$'tourn.randoms.miscommunication'[HPDFinal$SmoothingFactor=="12" & HPDFinal$'max.heur.number'=="20" & HPDFinal$'distance.type'=="manhattan" & HPDFinal$'distribution'=="poisson"]

MH20Manrug12Poistournb <- HPDFinal$'tourn.experts.miscommunication'[HPDFinal$SmoothingFactor=="12" & HPDFinal$'max.heur.number'=="20" & HPDFinal$'distance.type'=="manhattan" & HPDFinal$'distribution'=="poisson"]

t.test(MH20Manrug12Poistourna, MH20Manrug12Poistournb, paired=T)

MH20Manrug14Poistourna <- HPDFinal$'tourn.randoms.miscommunication'[HPDFinal$SmoothingFactor=="14" & HPDFinal$'max.heur.number'=="20" & HPDFinal$'distance.type'=="manhattan" & HPDFinal$'distribution'=="poisson"]

MH20Manrug14Poistournb <- HPDFinal$'tourn.experts.miscommunication'[HPDFinal$SmoothingFactor=="14" & HPDFinal$'max.heur.number'=="20" & HPDFinal$'distance.type'=="manhattan" & HPDFinal$'distribution'=="poisson"]

t.test(MH20Manrug14Poistourna, MH20Manrug14Poistournb, paired=T)

MH20Manrug16Poistourna <- HPDFinal$'tourn.randoms.miscommunication'[HPDFinal$SmoothingFactor=="16" & HPDFinal$'max.heur.number'=="20" & HPDFinal$'distance.type'=="manhattan" & HPDFinal$'distribution'=="poisson"]

MH20Manrug16Poistournb <- HPDFinal$'tourn.experts.miscommunication'[HPDFinal$SmoothingFactor=="16" & HPDFinal$'max.heur.number'=="20" & HPDFinal$'distance.type'=="manhattan" & HPDFinal$'distribution'=="poisson"]

t.test(MH20Manrug16Poistourna, MH20Manrug16Poistournb, paired=T)

MH20Manrug4Exprelaya <- HPDFinal$'relay.randoms.miscommunication'[HPDFinal$SmoothingFactor=="4" & HPDFinal$'max.heur.number'=="20" & HPDFinal$'distance.type'=="manhattan" & HPDFinal$'distribution'=="exponential"]

MH20Manrug4Exprelayb <- HPDFinal$'relay.experts.miscommunication'[HPDFinal$SmoothingFactor=="4" & HPDFinal$'max.heur.number'=="20" & HPDFinal$'distance.type'=="manhattan" & HPDFinal$'distribution'=="exponential"]

t.test(MH20Manrug4Exprelaya, MH20Manrug4Exprelayb, paired=T)

MH20Manrug6Exprelaya <- HPDFinal$'relay.randoms.miscommunication'[HPDFinal$SmoothingFactor=="6" & HPDFinal$'max.heur.number'=="20" & HPDFinal$'distance.type'=="manhattan" & HPDFinal$'distribution'=="exponential"]

MH20Manrug6Exprelayb <- HPDFinal$'relay.experts.miscommunication'[HPDFinal$SmoothingFactor=="6" & HPDFinal$'max.heur.number'=="20" & HPDFinal$'distance.type'=="manhattan" & HPDFinal$'distribution'=="exponential"]

t.test(MH20Manrug6Exprelaya, MH20Manrug6Exprelayb, paired=T)

MH20Manrug8Exprelaya <- HPDFinal$'relay.randoms.miscommunication'[HPDFinal$SmoothingFactor=="8" & HPDFinal$'max.heur.number'=="20" & HPDFinal$'distance.type'=="manhattan" & HPDFinal$'distribution'=="exponential"]

MH20Manrug8Exprelayb <- HPDFinal$'relay.experts.miscommunication'[HPDFinal$SmoothingFactor=="8" & HPDFinal$'max.heur.number'=="20" & HPDFinal$'distance.type'=="manhattan" & HPDFinal$'distribution'=="exponential"]

t.test(MH20Manrug8Exprelaya, MH20Manrug8Exprelayb, paired=T)

MH20Manrug10Exprelaya <- HPDFinal$'relay.randoms.miscommunication'[HPDFinal$SmoothingFactor=="10" & HPDFinal$'max.heur.number'=="20" & HPDFinal$'distance.type'=="manhattan" & HPDFinal$'distribution'=="exponential"]

MH20Manrug10Exprelayb <- HPDFinal$'relay.experts.miscommunication'[HPDFinal$SmoothingFactor=="10" & HPDFinal$'max.heur.number'=="20" & HPDFinal$'distance.type'=="manhattan" & HPDFinal$'distribution'=="exponential"]

t.test(MH20Manrug10Exprelaya, MH20Manrug10Exprelayb, paired=T)

MH20Manrug12Exprelaya <- HPDFinal$'relay.randoms.miscommunication'[HPDFinal$SmoothingFactor=="12" & HPDFinal$'max.heur.number'=="20" & HPDFinal$'distance.type'=="manhattan" & HPDFinal$'distribution'=="exponential"]

MH20Manrug12Exprelayb <- HPDFinal$'relay.experts.miscommunication'[HPDFinal$SmoothingFactor=="12" & HPDFinal$'max.heur.number'=="20" & HPDFinal$'distance.type'=="manhattan" & HPDFinal$'distribution'=="exponential"]

t.test(MH20Manrug12Exprelaya, MH20Manrug12Exprelayb, paired=T)

MH20Manrug14Exprelaya <- HPDFinal$'relay.randoms.miscommunication'[HPDFinal$SmoothingFactor=="14" & HPDFinal$'max.heur.number'=="20" & HPDFinal$'distance.type'=="manhattan" & HPDFinal$'distribution'=="exponential"]

MH20Manrug14Exprelayb <- HPDFinal$'relay.experts.miscommunication'[HPDFinal$SmoothingFactor=="14" & HPDFinal$'max.heur.number'=="20" & HPDFinal$'distance.type'=="manhattan" & HPDFinal$'distribution'=="exponential"]

t.test(MH20Manrug14Exprelaya, MH20Manrug14Exprelayb, paired=T)

MH20Manrug16Exprelaya <- HPDFinal$'relay.randoms.miscommunication'[HPDFinal$SmoothingFactor=="16" & HPDFinal$'max.heur.number'=="20" & HPDFinal$'distance.type'=="manhattan" & HPDFinal$'distribution'=="exponential"]

MH20Manrug16Exprelayb <- HPDFinal$'relay.experts.miscommunication'[HPDFinal$SmoothingFactor=="16" & HPDFinal$'max.heur.number'=="20" & HPDFinal$'distance.type'=="manhattan" & HPDFinal$'distribution'=="exponential"]

t.test(MH20Manrug12Exprelaya, MH20Manrug12Exprelayb, paired=T)

MH20Manrug4Exptourna <- HPDFinal$'tourn.randoms.miscommunication'[HPDFinal$SmoothingFactor=="4" & HPDFinal$'max.heur.number'=="20" & HPDFinal$'distance.type'=="manhattan" & HPDFinal$'distribution'=="exponential"]

MH20Manrug4Exptournb <- HPDFinal$'tourn.experts.miscommunication'[HPDFinal$SmoothingFactor=="4" & HPDFinal$'max.heur.number'=="20" & HPDFinal$'distance.type'=="manhattan" & HPDFinal$'distribution'=="exponential"]

t.test(MH20Manrug4Exptourna, MH20Manrug4Exptournb, paired=T)

MH20Manrug6Exptourna <- HPDFinal$'tourn.randoms.miscommunication'[HPDFinal$SmoothingFactor=="6" & HPDFinal$'max.heur.number'=="20" & HPDFinal$'distance.type'=="manhattan" & HPDFinal$'distribution'=="exponential"]

MH20Manrug6Exptournb <- HPDFinal$'tourn.experts.miscommunication'[HPDFinal$SmoothingFactor=="6" & HPDFinal$'max.heur.number'=="20" & HPDFinal$'distance.type'=="manhattan" & HPDFinal$'distribution'=="exponential"]

t.test(MH20Manrug6Exptourna, MH20Manrug6Exptournb, paired=T)

MH20Manrug8Exptourna <- HPDFinal$'tourn.randoms.miscommunication'[HPDFinal$SmoothingFactor=="8" & HPDFinal$'max.heur.number'=="20" & HPDFinal$'distance.type'=="manhattan" & HPDFinal$'distribution'=="exponential"]

MH20Manrug8Exptournb <- HPDFinal$'tourn.experts.miscommunication'[HPDFinal$SmoothingFactor=="8" & HPDFinal$'max.heur.number'=="20" & HPDFinal$'distance.type'=="manhattan" & HPDFinal$'distribution'=="exponential"]

t.test(MH20Manrug8Exptourna, MH20Manrug8Exptournb, paired=T)

MH20Manrug10Exptourna <- HPDFinal$'tourn.randoms.miscommunication'[HPDFinal$SmoothingFactor=="10" & HPDFinal$'max.heur.number'=="20" & HPDFinal$'distance.type'=="manhattan" & HPDFinal$'distribution'=="exponential"]

MH20Manrug10Exptournb <- HPDFinal$'tourn.experts.miscommunication'[HPDFinal$SmoothingFactor=="10" & HPDFinal$'max.heur.number'=="20" & HPDFinal$'distance.type'=="manhattan" & HPDFinal$'distribution'=="exponential"]

t.test(MH20Manrug10Exptourna, MH20Manrug10Exptournb, paired=T)

MH20Manrug12Exptourna <- HPDFinal$'tourn.randoms.miscommunication'[HPDFinal$SmoothingFactor=="12" & HPDFinal$'max.heur.number'=="20" & HPDFinal$'distance.type'=="manhattan" & HPDFinal$'distribution'=="exponential"]

MH20Manrug12Exptournb <- HPDFinal$'tourn.experts.miscommunication'[HPDFinal$SmoothingFactor=="12" & HPDFinal$'max.heur.number'=="20" & HPDFinal$'distance.type'=="manhattan" & HPDFinal$'distribution'=="exponential"]

t.test(MH20Manrug12Exptourna, MH20Manrug12Exptournb, paired=T)

MH20Manrug14Exptourna <- HPDFinal$'tourn.randoms.miscommunication'[HPDFinal$SmoothingFactor=="14" & HPDFinal$'max.heur.number'=="20" & HPDFinal$'distance.type'=="manhattan" & HPDFinal$'distribution'=="exponential"]

MH20Manrug14Exptournb <- HPDFinal$'tourn.experts.miscommunication'[HPDFinal$SmoothingFactor=="14" & HPDFinal$'max.heur.number'=="20" & HPDFinal$'distance.type'=="manhattan" & HPDFinal$'distribution'=="exponential"]

t.test(MH20Manrug14Exptourna, MH20Manrug14Exptournb, paired=T)

MH20Manrug16Exptourna <- HPDFinal$'tourn.randoms.miscommunication'[HPDFinal$SmoothingFactor=="16" & HPDFinal$'max.heur.number'=="20" & HPDFinal$'distance.type'=="manhattan" & HPDFinal$'distribution'=="exponential"]

MH20Manrug16Exptournb <- HPDFinal$'tourn.experts.miscommunication'[HPDFinal$SmoothingFactor=="16" & HPDFinal$'max.heur.number'=="20" & HPDFinal$'distance.type'=="manhattan" & HPDFinal$'distribution'=="exponential"]

t.test(MH20Manrug16Exptourna, MH20Manrug16Exptournb, paired=T)

MH20Manrug4Fixedrelaya <- HPDFinal$'relay.randoms.miscommunication.fixedsize'[HPDFinal$SmoothingFactor=="4" & HPDFinal$'max.heur.number'=="20" & HPDFinal$'distance.type'=="manhattan"]

MH20Manrug4Fixedrelayb <- HPDFinal$'relay.experts.miscommunication.fixedsize'[HPDFinal$SmoothingFactor=="4" & HPDFinal$'max.heur.number'=="20" & HPDFinal$'distance.type'=="manhattan"]

t.test(MH20Manrug4Fixedrelaya, MH20Manrug4Fixedrelayb, paired=T)

MH20Manrug6Fixedrelaya <- HPDFinal$'relay.randoms.miscommunication.fixedsize'[HPDFinal$SmoothingFactor=="6" & HPDFinal$'max.heur.number'=="20" & HPDFinal$'distance.type'=="manhattan"]

MH20Manrug6Fixedrelayb <- HPDFinal$'relay.experts.miscommunication.fixedsize'[HPDFinal$SmoothingFactor=="6" & HPDFinal$'max.heur.number'=="20" & HPDFinal$'distance.type'=="manhattan"]

t.test(MH20Manrug6Fixedrelaya, MH20Manrug6Fixedrelayb, paired=T)

MH20Manrug8Fixedrelaya <- HPDFinal$'relay.randoms.miscommunication.fixedsize'[HPDFinal$SmoothingFactor=="8" & HPDFinal$'max.heur.number'=="20" & HPDFinal$'distance.type'=="manhattan"]

MH20Manrug8Fixedrelayb <- HPDFinal$'relay.experts.miscommunication.fixedsize'[HPDFinal$SmoothingFactor=="8" & HPDFinal$'max.heur.number'=="20" & HPDFinal$'distance.type'=="manhattan"]

t.test(MH20Manrug8Fixedrelaya, MH20Manrug8Fixedrelayb, paired=T)

MH20Manrug10Fixedrelaya <- HPDFinal$'relay.randoms.miscommunication.fixedsize'[HPDFinal$SmoothingFactor=="10" & HPDFinal$'max.heur.number'=="20" & HPDFinal$'distance.type'=="manhattan"]

MH20Manrug10Fixedrelayb <- HPDFinal$'relay.experts.miscommunication.fixedsize'[HPDFinal$SmoothingFactor=="10" & HPDFinal$'max.heur.number'=="20" & HPDFinal$'distance.type'=="manhattan"]

t.test(MH20Manrug10Fixedrelaya, MH20Manrug10Fixedrelayb, paired=T)

MH20Manrug12Fixedrelaya <- HPDFinal$'relay.randoms.miscommunication.fixedsize'[HPDFinal$SmoothingFactor=="12" & HPDFinal$'max.heur.number'=="20" & HPDFinal$'distance.type'=="manhattan"]

MH20Manrug12Fixedrelayb <- HPDFinal$'relay.experts.miscommunication.fixedsize'[HPDFinal$SmoothingFactor=="12" & HPDFinal$'max.heur.number'=="20" & HPDFinal$'distance.type'=="manhattan"]

t.test(MH20Manrug12Fixedrelaya, MH20Manrug12Fixedrelayb, paired=T)

MH20Manrug14Fixedrelaya <- HPDFinal$'relay.randoms.miscommunication.fixedsize'[HPDFinal$SmoothingFactor=="14" & HPDFinal$'max.heur.number'=="20" & HPDFinal$'distance.type'=="manhattan"]

MH20Manrug14Fixedrelayb <- HPDFinal$'relay.experts.miscommunication.fixedsize'[HPDFinal$SmoothingFactor=="14" & HPDFinal$'max.heur.number'=="20" & HPDFinal$'distance.type'=="manhattan"]

t.test(MH20Manrug14Fixedrelaya, MH20Manrug14Fixedrelayb, paired=T)

MH20Manrug16Fixedrelaya <- HPDFinal$'relay.randoms.miscommunication.fixedsize'[HPDFinal$SmoothingFactor=="16" & HPDFinal$'max.heur.number'=="20" & HPDFinal$'distance.type'=="manhattan"]

MH20Manrug16Fixedrelayb <- HPDFinal$'relay.experts.miscommunication.fixedsize'[HPDFinal$SmoothingFactor=="16" & HPDFinal$'max.heur.number'=="20" & HPDFinal$'distance.type'=="manhattan"]

t.test(MH20Manrug16Fixedrelaya, MH20Manrug16Fixedrelayb, paired=T)

MH20Manrug4Fixedtourna <- HPDFinal$'tourn.randoms.miscommunication.fixedsize'[HPDFinal$SmoothingFactor=="4" & HPDFinal$'max.heur.number'=="20" & HPDFinal$'distance.type'=="manhattan"]

MH20Manrug4Fixedtournb <- HPDFinal$'tourn.experts.miscommunication.fixedsize'[HPDFinal$SmoothingFactor=="4" & HPDFinal$'max.heur.number'=="20" & HPDFinal$'distance.type'=="manhattan"]

t.test(MH20Manrug4Fixedtourna, MH20Manrug4Fixedtournb, paired=T)

MH20Manrug6Fixedtourna <- HPDFinal$'tourn.randoms.miscommunication.fixedsize'[HPDFinal$SmoothingFactor=="6" & HPDFinal$'max.heur.number'=="20" & HPDFinal$'distance.type'=="manhattan"]

MH20Manrug6Fixedtournb <- HPDFinal$'tourn.experts.miscommunication.fixedsize'[HPDFinal$SmoothingFactor=="6" & HPDFinal$'max.heur.number'=="20" & HPDFinal$'distance.type'=="manhattan"]

t.test(MH20Manrug6Fixedtourna, MH20Manrug6Fixedtournb, paired=T)

MH20Manrug8Fixedtourna <- HPDFinal$'tourn.randoms.miscommunication.fixedsize'[HPDFinal$SmoothingFactor=="8" & HPDFinal$'max.heur.number'=="20" & HPDFinal$'distance.type'=="manhattan"]

MH20Manrug8Fixedtournb <- HPDFinal$'tourn.experts.miscommunication.fixedsize'[HPDFinal$SmoothingFactor=="8" & HPDFinal$'max.heur.number'=="20" & HPDFinal$'distance.type'=="manhattan"]

t.test(MH20Manrug8Fixedtourna, MH20Manrug8Fixedtournb, paired=T)

MH20Manrug10Fixedtourna <- HPDFinal$'tourn.randoms.miscommunication.fixedsize'[HPDFinal$SmoothingFactor=="10" & HPDFinal$'max.heur.number'=="20" & HPDFinal$'distance.type'=="manhattan"]

MH20Manrug10Fixedtournb <- HPDFinal$'tourn.experts.miscommunication.fixedsize'[HPDFinal$SmoothingFactor=="10" & HPDFinal$'max.heur.number'=="20" & HPDFinal$'distance.type'=="manhattan"]

t.test(MH20Manrug10Fixedtourna, MH20Manrug10Fixedtournb, paired=T)

MH20Manrug12Fixedtourna <- HPDFinal$'tourn.randoms.miscommunication.fixedsize'[HPDFinal$SmoothingFactor=="12" & HPDFinal$'max.heur.number'=="20" & HPDFinal$'distance.type'=="manhattan"]

MH20Manrug12Fixedtournb <- HPDFinal$'tourn.experts.miscommunication.fixedsize'[HPDFinal$SmoothingFactor=="12" & HPDFinal$'max.heur.number'=="20" & HPDFinal$'distance.type'=="manhattan"]

t.test(MH20Manrug12Fixedtourna, MH20Manrug12Fixedtournb, paired=T)

MH20Manrug14Fixedtourna <- HPDFinal$'tourn.randoms.miscommunication.fixedsize'[HPDFinal$SmoothingFactor=="14" & HPDFinal$'max.heur.number'=="20" & HPDFinal$'distance.type'=="manhattan"]

MH20Manrug14Fixedtournb <- HPDFinal$'tourn.experts.miscommunication.fixedsize'[HPDFinal$SmoothingFactor=="14" & HPDFinal$'max.heur.number'=="20" & HPDFinal$'distance.type'=="manhattan"]

t.test(MH20Manrug14Fixedtourna, MH20Manrug14Fixedtournb, paired=T)

MH20Manrug16Fixedtourna <- HPDFinal$'tourn.randoms.miscommunication.fixedsize'[HPDFinal$SmoothingFactor=="16" & HPDFinal$'max.heur.number'=="20" & HPDFinal$'distance.type'=="manhattan"]

MH20Manrug16Fixedtournb <- HPDFinal$'tourn.experts.miscommunication.fixedsize'[HPDFinal$SmoothingFactor=="16" & HPDFinal$'max.heur.number'=="20" & HPDFinal$'distance.type'=="manhattan"]

t.test(MH20Manrug16Fixedtourna, MH20Manrug16Fixedtournb, paired=T)

**Hamming (match) Distance Measure**

MH20Hamrug4relaya <- HPDFinal$'relay.randoms'[HPDFinal$SmoothingFactor=="4" & HPDFinal$'max.heur.number'=="20"]

MH20Hamrug4relayb <- HPDFinal$'relay.experts'[HPDFinal$SmoothingFactor=="4" & HPDFinal$'max.heur.number'=="20"]

t.test(MH20Hamrug4relaya, MH20Hamrug4relayb, paired=T)

MH20Hamrug6relaya <- HPDFinal$'relay.randoms'[HPDFinal$SmoothingFactor=="6" & HPDFinal$'max.heur.number'=="20"]

MH20Hamrug6relayb <- HPDFinal$'relay.experts'[HPDFinal$SmoothingFactor=="6" & HPDFinal$'max.heur.number'=="20"]

t.test(MH20Hamrug6relaya, MH20Hamrug6relayb, paired=T)

MH20Hamrug8relaya <- HPDFinal$'relay.randoms'[HPDFinal$SmoothingFactor=="8" & HPDFinal$'max.heur.number'=="20"]

MH20Hamrug8relayb <- HPDFinal$'relay.experts'[HPDFinal$SmoothingFactor=="8" & HPDFinal$'max.heur.number'=="20"]

t.test(MH20Hamrug8relaya, MH20Hamrug8relayb, paired=T)

MH20Hamrug10relaya <- HPDFinal$'relay.randoms'[HPDFinal$SmoothingFactor=="10" & HPDFinal$'max.heur.number'=="20"]

MH20Hamrug10relayb <- HPDFinal$'relay.experts'[HPDFinal$SmoothingFactor=="10" & HPDFinal$'max.heur.number'=="20"]

t.test(MH20Hamrug10relaya, MH20Hamrug10relayb, paired=T)

MH20Hamrug12relaya <- HPDFinal$'relay.randoms'[HPDFinal$SmoothingFactor=="12" & HPDFinal$'max.heur.number'=="20"]

MH20Hamrug12relayb <- HPDFinal$'relay.experts'[HPDFinal$SmoothingFactor=="12" & HPDFinal$'max.heur.number'=="20"]

t.test(MH20Hamrug12relaya, MH20Hamrug12relayb, paired=T)

MH20Hamrug14relaya <- HPDFinal$'relay.randoms'[HPDFinal$SmoothingFactor=="14" & HPDFinal$'max.heur.number'=="20"]

MH20Hamrug14relayb <- HPDFinal$'relay.experts'[HPDFinal$SmoothingFactor=="14" & HPDFinal$'max.heur.number'=="20"]

t.test(MH20Hamrug14relaya, MH20Hamrug14relayb, paired=T)

MH20Hamrug16relaya <- HPDFinal$'relay.randoms'[HPDFinal$SmoothingFactor=="16" & HPDFinal$'max.heur.number'=="20"]

MH20Hamrug16relayb <- HPDFinal$'relay.experts'[HPDFinal$SmoothingFactor=="16" & HPDFinal$'max.heur.number'=="20"]

t.test(MH20Hamrug16relaya, MH20Hamrug16relayb, paired=T)

MH20Hamrug4tourna <- HPDFinal$'tourn.randoms'[HPDFinal$SmoothingFactor=="4" & HPDFinal$'max.heur.number'=="20"]

MH20Hamrug4tournb <- HPDFinal$'tourn.experts'[HPDFinal$SmoothingFactor=="4" & HPDFinal$'max.heur.number'=="20"]

t.test(MH20Hamrug4tourna, MH20Hamrug4tournb, paired=T)

MH20Hamrug6tourna <- HPDFinal$'tourn.randoms'[HPDFinal$SmoothingFactor=="6" & HPDFinal$'max.heur.number'=="20"]

MH20Hamrug6tournb <- HPDFinal$'tourn.experts'[HPDFinal$SmoothingFactor=="6" & HPDFinal$'max.heur.number'=="20"]

t.test(MH20Hamrug6tourna, MH20Hamrug6tournb, paired=T)

MH20Hamrug8tourna <- HPDFinal$'tourn.randoms'[HPDFinal$SmoothingFactor=="8" & HPDFinal$'max.heur.number'=="20"]

MH20Hamrug8tournb <- HPDFinal$'tourn.experts'[HPDFinal$SmoothingFactor=="8" & HPDFinal$'max.heur.number'=="20"]

t.test(MH20Hamrug8tourna, MH20Hamrug8tournb, paired=T)

MH20Hamrug10tourna <- HPDFinal$'tourn.randoms'[HPDFinal$SmoothingFactor=="10" & HPDFinal$'max.heur.number'=="20"]

MH20Hamrug10tournb <- HPDFinal$'tourn.experts'[HPDFinal$SmoothingFactor=="10" & HPDFinal$'max.heur.number'=="20"]

t.test(MH20Hamrug10tourna, MH20Hamrug10tournb, paired=T)

MH20Hamrug12tourna <- HPDFinal$'tourn.randoms'[HPDFinal$SmoothingFactor=="12" & HPDFinal$'max.heur.number'=="20"]

MH20Hamrug12tournb <- HPDFinal$'tourn.experts'[HPDFinal$SmoothingFactor=="12" & HPDFinal$'max.heur.number'=="20"]

t.test(MH20Hamrug12tourna, MH20Hamrug12tournb, paired=T)

MH20Hamrug14tourna <- HPDFinal$'tourn.randoms'[HPDFinal$SmoothingFactor=="14" & HPDFinal$'max.heur.number'=="20"]

MH20Hamrug14tournb <- HPDFinal$'tourn.experts'[HPDFinal$SmoothingFactor=="14" & HPDFinal$'max.heur.number'=="20"]

t.test(MH20Hamrug14tourna, MH20Hamrug14tournb, paired=T)

MH20Hamrug16tourna <- HPDFinal$'tourn.randoms'[HPDFinal$SmoothingFactor=="16" & HPDFinal$'max.heur.number'=="20"]

MH20Hamrug16tournb <- HPDFinal$'tourn.experts'[HPDFinal$SmoothingFactor=="16" & HPDFinal$'max.heur.number'=="20"]

t.test(MH20Hamrug16tourna, MH20Hamrug16tournb, paired=T)

MH20Hamrug4Normrelaya <- HPDFinal$'relay.randoms.miscommunication'[HPDFinal$SmoothingFactor=="4" & HPDFinal$'max.heur.number'=="20" & HPDFinal$'distance.type'=="hamming" & HPDFinal$'distribution'=="normal"]

MH20Hamrug4Normrelayb <- HPDFinal$'relay.experts.miscommunication'[HPDFinal$SmoothingFactor=="4" & HPDFinal$'max.heur.number'=="20" & HPDFinal$'distance.type'=="hamming" & HPDFinal$'distribution'=="normal"]

t.test(MH20Hamrug4Normrelaya, MH20Hamrug4Normrelayb, paired=T)

MH20Hamrug6Normrelaya <- HPDFinal$'relay.randoms.miscommunication'[HPDFinal$SmoothingFactor=="6" & HPDFinal$'max.heur.number'=="20" & HPDFinal$'distance.type'=="hamming" & HPDFinal$'distribution'=="normal"]

MH20Hamrug6Normrelayb <- HPDFinal$'relay.experts.miscommunication'[HPDFinal$SmoothingFactor=="6" & HPDFinal$'max.heur.number'=="20" & HPDFinal$'distance.type'=="hamming" & HPDFinal$'distribution'=="normal"]

t.test(MH20Hamrug6Normrelaya, MH20Hamrug6Normrelayb, paired=T)

MH20Hamrug8Normrelaya <- HPDFinal$'relay.randoms.miscommunication'[HPDFinal$SmoothingFactor=="8" & HPDFinal$'max.heur.number'=="20" & HPDFinal$'distance.type'=="hamming" & HPDFinal$'distribution'=="normal"]

MH20Hamrug8Normrelayb <- HPDFinal$'relay.experts.miscommunication'[HPDFinal$SmoothingFactor=="8" & HPDFinal$'max.heur.number'=="20" & HPDFinal$'distance.type'=="hamming" & HPDFinal$'distribution'=="normal"]

t.test(MH20Hamrug8Normrelaya, MH20Hamrug8Normrelayb, paired=T)

MH20Hamrug10Normrelaya <- HPDFinal$'relay.randoms.miscommunication'[HPDFinal$SmoothingFactor=="10" & HPDFinal$'max.heur.number'=="20" & HPDFinal$'distance.type'=="hamming" & HPDFinal$'distribution'=="normal"]

MH20Hamrug10Normrelayb <- HPDFinal$'relay.experts.miscommunication'[HPDFinal$SmoothingFactor=="10" & HPDFinal$'max.heur.number'=="20" & HPDFinal$'distance.type'=="hamming" & HPDFinal$'distribution'=="normal"]

t.test(MH20Hamrug10Normrelaya, MH20Hamrug10Normrelayb, paired=T)

MH20Hamrug12Normrelaya <- HPDFinal$'relay.randoms.miscommunication'[HPDFinal$SmoothingFactor=="12" & HPDFinal$'max.heur.number'=="20" & HPDFinal$'distance.type'=="hamming" & HPDFinal$'distribution'=="normal"]

MH20Hamrug12Normrelayb <- HPDFinal$'relay.experts.miscommunication'[HPDFinal$SmoothingFactor=="12" & HPDFinal$'max.heur.number'=="20" & HPDFinal$'distance.type'=="hamming" & HPDFinal$'distribution'=="normal"]

t.test(MH20Hamrug12Normrelaya, MH20Hamrug12Normrelayb, paired=T)

MH20Hamrug14Normrelaya <- HPDFinal$'relay.randoms.miscommunication'[HPDFinal$SmoothingFactor=="14" & HPDFinal$'max.heur.number'=="20" & HPDFinal$'distance.type'=="hamming" & HPDFinal$'distribution'=="normal"]

MH20Hamrug14Normrelayb <- HPDFinal$'relay.experts.miscommunication'[HPDFinal$SmoothingFactor=="14" & HPDFinal$'max.heur.number'=="20" & HPDFinal$'distance.type'=="hamming" & HPDFinal$'distribution'=="normal"]

t.test(MH20Hamrug14Normrelaya, MH20Hamrug14Normrelayb, paired=T)

MH20Hamrug16Normrelaya <- HPDFinal$'relay.randoms.miscommunication'[HPDFinal$SmoothingFactor=="16" & HPDFinal$'max.heur.number'=="20" & HPDFinal$'distance.type'=="hamming" & HPDFinal$'distribution'=="normal"]

MH20Hamrug16Normrelayb <- HPDFinal$'relay.experts.miscommunication'[HPDFinal$SmoothingFactor=="16" & HPDFinal$'max.heur.number'=="20" & HPDFinal$'distance.type'=="hamming" & HPDFinal$'distribution'=="normal"]

t.test(MH20Hamrug16Normrelaya, MH20Hamrug16Normrelayb, paired=T)

***

MH20Hamrug4Normtourna <- HPDFinal$'tourn.randoms.miscommunication'[HPDFinal$SmoothingFactor=="4" & HPDFinal$'max.heur.number'=="20" & HPDFinal$'distance.type'=="hamming" & HPDFinal$'distribution'=="normal"]

MH20Hamrug4Normtournb <- HPDFinal$'tourn.experts.miscommunication'[HPDFinal$SmoothingFactor=="4" & HPDFinal$'max.heur.number'=="20" & HPDFinal$'distance.type'=="hamming" & HPDFinal$'distribution'=="normal"]

t.test(MH20Hamrug4Normtourna, MH20Hamrug4Normtournb, paired=T)

MH20Hamrug6Normtourna <- HPDFinal$'tourn.randoms.miscommunication'[HPDFinal$SmoothingFactor=="6" & HPDFinal$'max.heur.number'=="20" & HPDFinal$'distance.type'=="hamming" & HPDFinal$'distribution'=="normal"]

MH20Hamrug6Normtournb <- HPDFinal$'tourn.experts.miscommunication'[HPDFinal$SmoothingFactor=="6" & HPDFinal$'max.heur.number'=="20" & HPDFinal$'distance.type'=="hamming" & HPDFinal$'distribution'=="normal"]

t.test(MH20Hamrug6Normtourna, MH20Hamrug6Normtournb, paired=T)

MH20Hamrug8Normtourna <- HPDFinal$'tourn.randoms.miscommunication'[HPDFinal$SmoothingFactor=="8" & HPDFinal$'max.heur.number'=="20" & HPDFinal$'distance.type'=="hamming" & HPDFinal$'distribution'=="normal"]

MH20Hamrug8Normtournb <- HPDFinal$'tourn.experts.miscommunication'[HPDFinal$SmoothingFactor=="8" & HPDFinal$'max.heur.number'=="20" & HPDFinal$'distance.type'=="hamming" & HPDFinal$'distribution'=="normal"]

t.test(MH20Hamrug8Normtourna, MH20Hamrug8Normtournb, paired=T)

MH20Hamrug10Normtourna <- HPDFinal$'tourn.randoms.miscommunication'[HPDFinal$SmoothingFactor=="10" & HPDFinal$'max.heur.number'=="20" & HPDFinal$'distance.type'=="hamming" & HPDFinal$'distribution'=="normal"]

MH20Hamrug10Normtournb <- HPDFinal$'tourn.experts.miscommunication'[HPDFinal$SmoothingFactor=="10" & HPDFinal$'max.heur.number'=="20" & HPDFinal$'distance.type'=="hamming" & HPDFinal$'distribution'=="normal"]

t.test(MH20Hamrug10Normtourna, MH20Hamrug10Normtournb, paired=T)

MH20Hamrug12Normtourna <- HPDFinal$'tourn.randoms.miscommunication'[HPDFinal$SmoothingFactor=="12" & HPDFinal$'max.heur.number'=="20" & HPDFinal$'distance.type'=="hamming" & HPDFinal$'distribution'=="normal"]

MH20Hamrug12Normtournb <- HPDFinal$'tourn.experts.miscommunication'[HPDFinal$SmoothingFactor=="12" & HPDFinal$'max.heur.number'=="20" & HPDFinal$'distance.type'=="hamming" & HPDFinal$'distribution'=="normal"]

t.test(MH20Hamrug12Normtourna, MH20Hamrug12Normtournb, paired=T)

MH20Hamrug14Normtourna <- HPDFinal$'tourn.randoms.miscommunication'[HPDFinal$SmoothingFactor=="14" & HPDFinal$'max.heur.number'=="20" & HPDFinal$'distance.type'=="hamming" & HPDFinal$'distribution'=="normal"]

MH20Hamrug14Normtournb <- HPDFinal$'tourn.experts.miscommunication'[HPDFinal$SmoothingFactor=="14" & HPDFinal$'max.heur.number'=="20" & HPDFinal$'distance.type'=="hamming" & HPDFinal$'distribution'=="normal"]

t.test(MH20Hamrug14Normtourna, MH20Hamrug14Normtournb, paired=T)

MH20Hamrug16Normtourna <- HPDFinal$'tourn.randoms.miscommunication'[HPDFinal$SmoothingFactor=="16" & HPDFinal$'max.heur.number'=="20" & HPDFinal$'distance.type'=="hamming" & HPDFinal$'distribution'=="normal"]

MH20Hamrug16Normtournb <- HPDFinal$'tourn.experts.miscommunication'[HPDFinal$SmoothingFactor=="16" & HPDFinal$'max.heur.number'=="20" & HPDFinal$'distance.type'=="hamming" & HPDFinal$'distribution'=="normal"]

t.test(MH20Hamrug16Normtourna, MH20Hamrug16Normtournb, paired=T)

***

MH20Hamrug4Poisrelaya <- HPDFinal$'relay.randoms.miscommunication'[HPDFinal$SmoothingFactor=="4" & HPDFinal$'max.heur.number'=="20" & HPDFinal$'distance.type'=="hamming" & HPDFinal$'distribution'=="poisson"]

MH20Hamrug4Poisrelayb <- HPDFinal$'relay.experts.miscommunication'[HPDFinal$SmoothingFactor=="4" & HPDFinal$'max.heur.number'=="20" & HPDFinal$'distance.type'=="hamming" & HPDFinal$'distribution'=="poisson"]

t.test(MH20Hamrug4Poisrelaya, MH20Hamrug4Poisrelayb, paired=T)

MH20Hamrug6Poisrelaya <- HPDFinal$'relay.randoms.miscommunication'[HPDFinal$SmoothingFactor=="6" & HPDFinal$'max.heur.number'=="20" & HPDFinal$'distance.type'=="hamming" & HPDFinal$'distribution'=="poisson"]

MH20Hamrug6Poisrelayb <- HPDFinal$'relay.experts.miscommunication'[HPDFinal$SmoothingFactor=="6" & HPDFinal$'max.heur.number'=="20" & HPDFinal$'distance.type'=="hamming" & HPDFinal$'distribution'=="poisson"]

t.test(MH20Hamrug6Poisrelaya, MH20Hamrug6Poisrelayb, paired=T)

MH20Hamrug8Poisrelaya <- HPDFinal$'relay.randoms.miscommunication'[HPDFinal$SmoothingFactor=="8" & HPDFinal$'max.heur.number'=="20" & HPDFinal$'distance.type'=="hamming" & HPDFinal$'distribution'=="poisson"]

MH20Hamrug8Poisrelayb <- HPDFinal$'relay.experts.miscommunication'[HPDFinal$SmoothingFactor=="8" & HPDFinal$'max.heur.number'=="20" & HPDFinal$'distance.type'=="hamming" & HPDFinal$'distribution'=="poisson"]

t.test(MH20Hamrug8Poisrelaya, MH20Hamrug8Poisrelayb, paired=T)

MH20Hamrug10Poisrelaya <- HPDFinal$'relay.randoms.miscommunication'[HPDFinal$SmoothingFactor=="10" & HPDFinal$'max.heur.number'=="20" & HPDFinal$'distance.type'=="hamming" & HPDFinal$'distribution'=="poisson"]

MH20Hamrug10Poisrelayb <- HPDFinal$'relay.experts.miscommunication'[HPDFinal$SmoothingFactor=="10" & HPDFinal$'max.heur.number'=="20" & HPDFinal$'distance.type'=="hamming" & HPDFinal$'distribution'=="poisson"]

t.test(MH20Hamrug10Poisrelaya, MH20Hamrug10Poisrelayb, paired=T)

MH20Hamrug12Poisrelaya <- HPDFinal$'relay.randoms.miscommunication'[HPDFinal$SmoothingFactor=="12" & HPDFinal$'max.heur.number'=="20" & HPDFinal$'distance.type'=="hamming" & HPDFinal$'distribution'=="poisson"]

MH20Hamrug12Poisrelayb <- HPDFinal$'relay.experts.miscommunication'[HPDFinal$SmoothingFactor=="12" & HPDFinal$'max.heur.number'=="20" & HPDFinal$'distance.type'=="hamming" & HPDFinal$'distribution'=="poisson"]

t.test(MH20Hamrug12Poisrelaya, MH20Hamrug12Poisrelayb, paired=T)

MH20Hamrug14Poisrelaya <- HPDFinal$'relay.randoms.miscommunication'[HPDFinal$SmoothingFactor=="14" & HPDFinal$'max.heur.number'=="20" & HPDFinal$'distance.type'=="hamming" & HPDFinal$'distribution'=="poisson"]

MH20Hamrug14Poisrelayb <- HPDFinal$'relay.experts.miscommunication'[HPDFinal$SmoothingFactor=="14" & HPDFinal$'max.heur.number'=="20" & HPDFinal$'distance.type'=="hamming" & HPDFinal$'distribution'=="poisson"]

t.test(MH20Hamrug14Poisrelaya, MH20Hamrug14Poisrelayb, paired=T)

MH20Hamrug16Poisrelaya <- HPDFinal$'relay.randoms.miscommunication'[HPDFinal$SmoothingFactor=="16" & HPDFinal$'max.heur.number'=="20" & HPDFinal$'distance.type'=="hamming" & HPDFinal$'distribution'=="poisson"]

MH20Hamrug16Poisrelayb <- HPDFinal$'relay.experts.miscommunication'[HPDFinal$SmoothingFactor=="16" & HPDFinal$'max.heur.number'=="20" & HPDFinal$'distance.type'=="hamming" & HPDFinal$'distribution'=="poisson"]

t.test(MH20Hamrug16Poisrelaya, MH20Hamrug16Poisrelayb, paired=T)

***

MH20Hamrug4Poistourna <- HPDFinal$'tourn.randoms.miscommunication'[HPDFinal$SmoothingFactor=="4" & HPDFinal$'max.heur.number'=="20" & HPDFinal$'distance.type'=="hamming" & HPDFinal$'distribution'=="poisson"]

MH20Hamrug4Poistournb <- HPDFinal$'tourn.experts.miscommunication'[HPDFinal$SmoothingFactor=="4" & HPDFinal$'max.heur.number'=="20" & HPDFinal$'distance.type'=="hamming" & HPDFinal$'distribution'=="poisson"]

t.test(MH20Hamrug4Poistourna, MH20Hamrug4Poistournb, paired=T)

MH20Hamrug6Poistourna <- HPDFinal$'tourn.randoms.miscommunication'[HPDFinal$SmoothingFactor=="6" & HPDFinal$'max.heur.number'=="20" & HPDFinal$'distance.type'=="hamming" & HPDFinal$'distribution'=="poisson"]

MH20Hamrug6Poistournb <- HPDFinal$'tourn.experts.miscommunication'[HPDFinal$SmoothingFactor=="6" & HPDFinal$'max.heur.number'=="20" & HPDFinal$'distance.type'=="hamming" & HPDFinal$'distribution'=="poisson"]

t.test(MH20Hamrug6Poistourna, MH20Hamrug6Poistournb, paired=T)

MH20Hamrug8Poistourna <- HPDFinal$'tourn.randoms.miscommunication'[HPDFinal$SmoothingFactor=="8" & HPDFinal$'max.heur.number'=="20" & HPDFinal$'distance.type'=="hamming" & HPDFinal$'distribution'=="poisson"]

MH20Hamrug8Poistournb <- HPDFinal$'tourn.experts.miscommunication'[HPDFinal$SmoothingFactor=="8" & HPDFinal$'max.heur.number'=="20" & HPDFinal$'distance.type'=="hamming" & HPDFinal$'distribution'=="poisson"]

t.test(MH20Hamrug6Poistourna, MH20Hamrug6Poistournb, paired=T)

MH20Hamrug10Poistourna <- HPDFinal$'tourn.randoms.miscommunication'[HPDFinal$SmoothingFactor=="10" & HPDFinal$'max.heur.number'=="20" & HPDFinal$'distance.type'=="hamming" & HPDFinal$'distribution'=="poisson"]

MH20Hamrug10Poistournb <- HPDFinal$'tourn.experts.miscommunication'[HPDFinal$SmoothingFactor=="10" & HPDFinal$'max.heur.number'=="20" & HPDFinal$'distance.type'=="hamming" & HPDFinal$'distribution'=="poisson"]

t.test(MH20Hamrug10Poistourna, MH20Hamrug10Poistournb, paired=T)

MH20Hamrug12Poistourna <- HPDFinal$'tourn.randoms.miscommunication'[HPDFinal$SmoothingFactor=="12" & HPDFinal$'max.heur.number'=="20" & HPDFinal$'distance.type'=="hamming" & HPDFinal$'distribution'=="poisson"]

MH20Hamrug12Poistournb <- HPDFinal$'tourn.experts.miscommunication'[HPDFinal$SmoothingFactor=="12" & HPDFinal$'max.heur.number'=="20" & HPDFinal$'distance.type'=="hamming" & HPDFinal$'distribution'=="poisson"]

t.test(MH20Hamrug12Poistourna, MH20Hamrug12Poistournb, paired=T)

MH20Hamrug14Poistourna <- HPDFinal$'tourn.randoms.miscommunication'[HPDFinal$SmoothingFactor=="14" & HPDFinal$'max.heur.number'=="20" & HPDFinal$'distance.type'=="hamming" & HPDFinal$'distribution'=="poisson"]

MH20Hamrug14Poistournb <- HPDFinal$'tourn.experts.miscommunication'[HPDFinal$SmoothingFactor=="14" & HPDFinal$'max.heur.number'=="20" & HPDFinal$'distance.type'=="hamming" & HPDFinal$'distribution'=="poisson"]

t.test(MH20Hamrug14Poistourna, MH20Hamrug14Poistournb, paired=T)

MH20Hamrug16Poistourna <- HPDFinal$'tourn.randoms.miscommunication'[HPDFinal$SmoothingFactor=="16" & HPDFinal$'max.heur.number'=="20" & HPDFinal$'distance.type'=="hamming" & HPDFinal$'distribution'=="poisson"]

MH20Hamrug16Poistournb <- HPDFinal$'tourn.experts.miscommunication'[HPDFinal$SmoothingFactor=="16" & HPDFinal$'max.heur.number'=="20" & HPDFinal$'distance.type'=="hamming" & HPDFinal$'distribution'=="poisson"]

t.test(MH20Hamrug16Poistourna, MH20Hamrug16Poistournb, paired=T)

***

MH20Hamrug4Exprelaya <- HPDFinal$'relay.randoms.miscommunication'[HPDFinal$SmoothingFactor=="4" & HPDFinal$'max.heur.number'=="20" & HPDFinal$'distance.type'=="hamming" & HPDFinal$'distribution'=="exponential"]

MH20Hamrug4Exprelayb <- HPDFinal$'relay.experts.miscommunication'[HPDFinal$SmoothingFactor=="4" & HPDFinal$'max.heur.number'=="20" & HPDFinal$'distance.type'=="hamming" & HPDFinal$'distribution'=="exponential"]

t.test(MH20Hamrug4Exprelaya, MH20Hamrug4Exprelayb, paired=T)

MH20Hamrug6Exprelaya <- HPDFinal$'relay.randoms.miscommunication'[HPDFinal$SmoothingFactor=="6" & HPDFinal$'max.heur.number'=="20" & HPDFinal$'distance.type'=="hamming" & HPDFinal$'distribution'=="exponential"]

MH20Hamrug6Exprelayb <- HPDFinal$'relay.experts.miscommunication'[HPDFinal$SmoothingFactor=="6" & HPDFinal$'max.heur.number'=="20" & HPDFinal$'distance.type'=="hamming" & HPDFinal$'distribution'=="exponential"]

t.test(MH20Hamrug6Exprelaya, MH20Hamrug6Exprelayb, paired=T)

MH20Hamrug8Exprelaya <- HPDFinal$'relay.randoms.miscommunication'[HPDFinal$SmoothingFactor=="8" & HPDFinal$'max.heur.number'=="20" & HPDFinal$'distance.type'=="hamming" & HPDFinal$'distribution'=="exponential"]

MH20Hamrug8Exprelayb <- HPDFinal$'relay.experts.miscommunication'[HPDFinal$SmoothingFactor=="8" & HPDFinal$'max.heur.number'=="20" & HPDFinal$'distance.type'=="hamming" & HPDFinal$'distribution'=="exponential"]

t.test(MH20Hamrug8Exprelaya, MH20Hamrug8Exprelayb, paired=T)

MH20Hamrug10Exprelaya <- HPDFinal$'relay.randoms.miscommunication'[HPDFinal$SmoothingFactor=="10" & HPDFinal$'max.heur.number'=="20" & HPDFinal$'distance.type'=="hamming" & HPDFinal$'distribution'=="exponential"]

MH20Hamrug10Exprelayb <- HPDFinal$'relay.experts.miscommunication'[HPDFinal$SmoothingFactor=="10" & HPDFinal$'max.heur.number'=="20" & HPDFinal$'distance.type'=="hamming" & HPDFinal$'distribution'=="exponential"]

t.test(MH20Hamrug10Exprelaya, MH20Hamrug10Exprelayb, paired=T)

MH20Hamrug12Exprelaya <- HPDFinal$'relay.randoms.miscommunication'[HPDFinal$SmoothingFactor=="12" & HPDFinal$'max.heur.number'=="20" & HPDFinal$'distance.type'=="hamming" & HPDFinal$'distribution'=="exponential"]

MH20Hamrug12Exprelayb <- HPDFinal$'relay.experts.miscommunication'[HPDFinal$SmoothingFactor=="12" & HPDFinal$'max.heur.number'=="20" & HPDFinal$'distance.type'=="hamming" & HPDFinal$'distribution'=="exponential"]

t.test(MH20Hamrug12Exprelaya, MH20Hamrug12Exprelayb, paired=T)

MH20Hamrug14Exprelaya <- HPDFinal$'relay.randoms.miscommunication'[HPDFinal$SmoothingFactor=="14" & HPDFinal$'max.heur.number'=="20" & HPDFinal$'distance.type'=="hamming" & HPDFinal$'distribution'=="exponential"]

MH20Hamrug14Exprelayb <- HPDFinal$'relay.experts.miscommunication'[HPDFinal$SmoothingFactor=="14" & HPDFinal$'max.heur.number'=="20" & HPDFinal$'distance.type'=="hamming" & HPDFinal$'distribution'=="exponential"]

t.test(MH20Hamrug14Exprelaya, MH20Hamrug14Exprelayb, paired=T)

MH20Hamrug16Exprelaya <- HPDFinal$'relay.randoms.miscommunication'[HPDFinal$SmoothingFactor=="16" & HPDFinal$'max.heur.number'=="20" & HPDFinal$'distance.type'=="hamming" & HPDFinal$'distribution'=="exponential"]

MH20Hamrug16Exprelayb <- HPDFinal$'relay.experts.miscommunication'[HPDFinal$SmoothingFactor=="16" & HPDFinal$'max.heur.number'=="20" & HPDFinal$'distance.type'=="hamming" & HPDFinal$'distribution'=="exponential"]

t.test(MH20Hamrug16Exprelaya, MH20Hamrug16Exprelayb, paired=T)

***

MH20Hamrug4Exptourna <- HPDFinal$'tourn.randoms.miscommunication'[HPDFinal$SmoothingFactor=="4" & HPDFinal$'max.heur.number'=="20" & HPDFinal$'distance.type'=="hamming" & HPDFinal$'distribution'=="exponential"]

MH20Hamrug4Exptournb <- HPDFinal$'tourn.experts.miscommunication'[HPDFinal$SmoothingFactor=="4" & HPDFinal$'max.heur.number'=="20" & HPDFinal$'distance.type'=="hamming" & HPDFinal$'distribution'=="exponential"]

t.test(MH20Hamrug4Exptourna, MH20Hamrug4Exptournb, paired=T)

MH20Hamrug6Exptourna <- HPDFinal$'tourn.randoms.miscommunication'[HPDFinal$SmoothingFactor=="6" & HPDFinal$'max.heur.number'=="20" & HPDFinal$'distance.type'=="hamming" & HPDFinal$'distribution'=="exponential"]

MH20Hamrug6Exptournb <- HPDFinal$'tourn.experts.miscommunication'[HPDFinal$SmoothingFactor=="6" & HPDFinal$'max.heur.number'=="20" & HPDFinal$'distance.type'=="hamming" & HPDFinal$'distribution'=="exponential"]

t.test(MH20Hamrug6Exptourna, MH20Hamrug6Exptournb, paired=T)

MH20Hamrug8Exptourna <- HPDFinal$'tourn.randoms.miscommunication'[HPDFinal$SmoothingFactor=="8" & HPDFinal$'max.heur.number'=="20" & HPDFinal$'distance.type'=="hamming" & HPDFinal$'distribution'=="exponential"]

MH20Hamrug8Exptournb <- HPDFinal$'tourn.experts.miscommunication'[HPDFinal$SmoothingFactor=="8" & HPDFinal$'max.heur.number'=="20" & HPDFinal$'distance.type'=="hamming" & HPDFinal$'distribution'=="exponential"]

t.test(MH20Hamrug8Exptourna, MH20Hamrug8Exptournb, paired=T)

MH20Hamrug10Exptourna <- HPDFinal$'tourn.randoms.miscommunication'[HPDFinal$SmoothingFactor=="10" & HPDFinal$'max.heur.number'=="20" & HPDFinal$'distance.type'=="hamming" & HPDFinal$'distribution'=="exponential"]

MH20Hamrug10Exptournb <- HPDFinal$'tourn.experts.miscommunication'[HPDFinal$SmoothingFactor=="10" & HPDFinal$'max.heur.number'=="20" & HPDFinal$'distance.type'=="hamming" & HPDFinal$'distribution'=="exponential"]

t.test(MH20Hamrug10Exptourna, MH20Hamrug10Exptournb, paired=T)

MH20Hamrug12Exptourna <- HPDFinal$'tourn.randoms.miscommunication'[HPDFinal$SmoothingFactor=="12" & HPDFinal$'max.heur.number'=="20" & HPDFinal$'distance.type'=="hamming" & HPDFinal$'distribution'=="exponential"]

MH20Hamrug12Exptournb <- HPDFinal$'tourn.experts.miscommunication'[HPDFinal$SmoothingFactor=="12" & HPDFinal$'max.heur.number'=="20" & HPDFinal$'distance.type'=="hamming" & HPDFinal$'distribution'=="exponential"]

t.test(MH20Hamrug12Exptourna, MH20Hamrug12Exptournb, paired=T)

MH20Hamrug14Exptourna <- HPDFinal$'tourn.randoms.miscommunication'[HPDFinal$SmoothingFactor=="14" & HPDFinal$'max.heur.number'=="20" & HPDFinal$'distance.type'=="hamming" & HPDFinal$'distribution'=="exponential"]

MH20Hamrug14Exptournb <- HPDFinal$'tourn.experts.miscommunication'[HPDFinal$SmoothingFactor=="14" & HPDFinal$'max.heur.number'=="20" & HPDFinal$'distance.type'=="hamming" & HPDFinal$'distribution'=="exponential"]

t.test(MH20Hamrug14Exptourna, MH20Hamrug14Exptournb, paired=T)

MH20Hamrug16Exptourna <- HPDFinal$'tourn.randoms.miscommunication'[HPDFinal$SmoothingFactor=="16" & HPDFinal$'max.heur.number'=="20" & HPDFinal$'distance.type'=="hamming" & HPDFinal$'distribution'=="exponential"]

MH20Hamrug16Exptournb <- HPDFinal$'tourn.experts.miscommunication'[HPDFinal$SmoothingFactor=="16" & HPDFinal$'max.heur.number'=="20" & HPDFinal$'distance.type'=="hamming" & HPDFinal$'distribution'=="exponential"]

t.test(MH20Hamrug16Exptourna, MH20Hamrug16Exptournb, paired=T)

***

MH20Hamrug4Fixedrelaya <- HPDFinal$'relay.randoms.miscommunication.fixedsize'[HPDFinal$SmoothingFactor=="4" & HPDFinal$'max.heur.number'=="20" & HPDFinal$'distance.type'=="hamming"]

MH20Hamrug4Fixedrelayb <- HPDFinal$'relay.experts.miscommunication.fixedsize'[HPDFinal$SmoothingFactor=="4" & HPDFinal$'max.heur.number'=="20" & HPDFinal$'distance.type'=="hamming"]

t.test(MH20Hamrug4Fixedrelaya, MH20Hamrug4Fixedrelayb, paired=T)

MH20Hamrug6Fixedrelaya <- HPDFinal$'relay.randoms.miscommunication.fixedsize'[HPDFinal$SmoothingFactor=="6" & HPDFinal$'max.heur.number'=="20" & HPDFinal$'distance.type'=="hamming"]

MH20Hamrug6Fixedrelayb <- HPDFinal$'relay.experts.miscommunication.fixedsize'[HPDFinal$SmoothingFactor=="6" & HPDFinal$'max.heur.number'=="20" & HPDFinal$'distance.type'=="hamming"]

t.test(MH20Hamrug6Fixedrelaya, MH20Hamrug6Fixedrelayb, paired=T)

MH20Hamrug8Fixedrelaya <- HPDFinal$'relay.randoms.miscommunication.fixedsize'[HPDFinal$SmoothingFactor=="8" & HPDFinal$'max.heur.number'=="20" & HPDFinal$'distance.type'=="hamming"]

MH20Hamrug8Fixedrelayb <- HPDFinal$'relay.experts.miscommunication.fixedsize'[HPDFinal$SmoothingFactor=="8" & HPDFinal$'max.heur.number'=="20" & HPDFinal$'distance.type'=="hamming"]

t.test(MH20Hamrug8Fixedrelaya, MH20Hamrug8Fixedrelayb, paired=T)

MH20Hamrug10Fixedrelaya <- HPDFinal$'relay.randoms.miscommunication.fixedsize'[HPDFinal$SmoothingFactor=="10" & HPDFinal$'max.heur.number'=="20" & HPDFinal$'distance.type'=="hamming"]

MH20Hamrug10Fixedrelayb <- HPDFinal$'relay.experts.miscommunication.fixedsize'[HPDFinal$SmoothingFactor=="10" & HPDFinal$'max.heur.number'=="20" & HPDFinal$'distance.type'=="hamming"]

t.test(MH20Hamrug10Fixedrelaya, MH20Hamrug10Fixedrelayb, paired=T)

MH20Hamrug12Fixedrelaya <- HPDFinal$'relay.randoms.miscommunication.fixedsize'[HPDFinal$SmoothingFactor=="12" & HPDFinal$'max.heur.number'=="20" & HPDFinal$'distance.type'=="hamming"]

MH20Hamrug12Fixedrelayb <- HPDFinal$'relay.experts.miscommunication.fixedsize'[HPDFinal$SmoothingFactor=="12" & HPDFinal$'max.heur.number'=="20" & HPDFinal$'distance.type'=="hamming"]

t.test(MH20Hamrug12Fixedrelaya, MH20Hamrug12Fixedrelayb, paired=T)

MH20Hamrug14Fixedrelaya <- HPDFinal$'relay.randoms.miscommunication.fixedsize'[HPDFinal$SmoothingFactor=="14" & HPDFinal$'max.heur.number'=="20" & HPDFinal$'distance.type'=="hamming"]

MH20Hamrug14Fixedrelayb <- HPDFinal$'relay.experts.miscommunication.fixedsize'[HPDFinal$SmoothingFactor=="14" & HPDFinal$'max.heur.number'=="20" & HPDFinal$'distance.type'=="hamming"]

t.test(MH20Hamrug14Fixedrelaya, MH20Hamrug14Fixedrelayb, paired=T)

MH20Hamrug16Fixedrelaya <- HPDFinal$'relay.randoms.miscommunication.fixedsize'[HPDFinal$SmoothingFactor=="16" & HPDFinal$'max.heur.number'=="20" & HPDFinal$'distance.type'=="hamming"]

MH20Hamrug16Fixedrelayb <- HPDFinal$'relay.experts.miscommunication.fixedsize'[HPDFinal$SmoothingFactor=="16" & HPDFinal$'max.heur.number'=="20" & HPDFinal$'distance.type'=="hamming"]

t.test(MH20Hamrug16Fixedrelaya, MH20Hamrug16Fixedrelayb, paired=T)

***

MH20Hamrug4Fixedtourna <- HPDFinal$'tourn.randoms.miscommunication.fixedsize'[HPDFinal$SmoothingFactor=="4" & HPDFinal$'max.heur.number'=="20" & HPDFinal$'distance.type'=="hamming"]

MH20Hamrug4Fixedtournb <- HPDFinal$'tourn.experts.miscommunication.fixedsize'[HPDFinal$SmoothingFactor=="4" & HPDFinal$'max.heur.number'=="20" & HPDFinal$'distance.type'=="hamming"]

t.test(MH20Hamrug4Fixedtourna, MH20Hamrug4Fixedtournb, paired=T)

MH20Hamrug6Fixedtourna <- HPDFinal$'tourn.randoms.miscommunication.fixedsize'[HPDFinal$SmoothingFactor=="6" & HPDFinal$'max.heur.number'=="20" & HPDFinal$'distance.type'=="hamming"]

MH20Hamrug6Fixedtournb <- HPDFinal$'tourn.experts.miscommunication.fixedsize'[HPDFinal$SmoothingFactor=="6" & HPDFinal$'max.heur.number'=="20" & HPDFinal$'distance.type'=="hamming"]

t.test(MH20Hamrug6Fixedtourna, MH20Hamrug6Fixedtournb, paired=T)

MH20Hamrug8Fixedtourna <- HPDFinal$'tourn.randoms.miscommunication.fixedsize'[HPDFinal$SmoothingFactor=="8" & HPDFinal$'max.heur.number'=="20" & HPDFinal$'distance.type'=="hamming"]

MH20Hamrug8Fixedtournb <- HPDFinal$'tourn.experts.miscommunication.fixedsize'[HPDFinal$SmoothingFactor=="8" & HPDFinal$'max.heur.number'=="20" & HPDFinal$'distance.type'=="hamming"]

t.test(MH20Hamrug8Fixedtourna, MH20Hamrug8Fixedtournb, paired=T)

MH20Hamrug10Fixedtourna <- HPDFinal$'tourn.randoms.miscommunication.fixedsize'[HPDFinal$SmoothingFactor=="10" & HPDFinal$'max.heur.number'=="20" & HPDFinal$'distance.type'=="hamming"]

MH20Hamrug10Fixedtournb <- HPDFinal$'tourn.experts.miscommunication.fixedsize'[HPDFinal$SmoothingFactor=="10" & HPDFinal$'max.heur.number'=="20" & HPDFinal$'distance.type'=="hamming"]

t.test(MH20Hamrug10Fixedtourna, MH20Hamrug10Fixedtournb, paired=T)

MH20Hamrug12Fixedtourna <- HPDFinal$'tourn.randoms.miscommunication.fixedsize'[HPDFinal$SmoothingFactor=="12" & HPDFinal$'max.heur.number'=="20" & HPDFinal$'distance.type'=="hamming"]

MH20Hamrug12Fixedtournb <- HPDFinal$'tourn.experts.miscommunication.fixedsize'[HPDFinal$SmoothingFactor=="12" & HPDFinal$'max.heur.number'=="20" & HPDFinal$'distance.type'=="hamming"]

t.test(MH20Hamrug12Fixedtourna, MH20Hamrug12Fixedtournb, paired=T)

MH20Hamrug14Fixedtourna <- HPDFinal$'tourn.randoms.miscommunication.fixedsize'[HPDFinal$SmoothingFactor=="14" & HPDFinal$'max.heur.number'=="20" & HPDFinal$'distance.type'=="hamming"]

MH20Hamrug14Fixedtournb <- HPDFinal$'tourn.experts.miscommunication.fixedsize'[HPDFinal$SmoothingFactor=="14" & HPDFinal$'max.heur.number'=="20" & HPDFinal$'distance.type'=="hamming"]

t.test(MH20Hamrug14Fixedtourna, MH20Hamrug14Fixedtournb, paired=T)

MH20Hamrug16Fixedtourna <- HPDFinal$'tourn.randoms.miscommunication.fixedsize'[HPDFinal$SmoothingFactor=="16" & HPDFinal$'max.heur.number'=="20" & HPDFinal$'distance.type'=="hamming"]

MH20Hamrug16Fixedtournb <- HPDFinal$'tourn.experts.miscommunication.fixedsize'[HPDFinal$SmoothingFactor=="16" & HPDFinal$'max.heur.number'=="20" & HPDFinal$'distance.type'=="hamming"]

t.test(MH20Hamrug16Fixedtourna, MH20Hamrug16Fixedtournb, paired=T)

**Hybrid Hamming**

MH20Hamrug4tournrelaya <- HPDFinal$'tourn.of.relays.randoms'[HPDFinal$SmoothingFactor=="4" & HPDFinal$'max.heur.number'=="20"]

MH20Hamrug4tournrelayb <- HPDFinal$'tourn.of.relays.experts'[HPDFinal$SmoothingFactor=="4" & HPDFinal$'max.heur.number'=="20"]

t.test(MH20Hamrug4tournrelaya, MH20Hamrug4tournrelayb, paired=T)

MH20Hamrug6tournrelaya <- HPDFinal$'tourn.of.relays.randoms'[HPDFinal$SmoothingFactor=="6" & HPDFinal$'max.heur.number'=="20"]

MH20Hamrug6tournrelayb <- HPDFinal$'tourn.of.relays.experts'[HPDFinal$SmoothingFactor=="6" & HPDFinal$'max.heur.number'=="20"]

t.test(MH20Hamrug6tournrelaya, MH20Hamrug6tournrelayb, paired=T)

MH20Hamrug8tournrelaya <- HPDFinal$'tourn.of.relays.randoms'[HPDFinal$SmoothingFactor=="8" & HPDFinal$'max.heur.number'=="20"]

MH20Hamrug8tournrelayb <- HPDFinal$'tourn.of.relays.experts'[HPDFinal$SmoothingFactor=="8" & HPDFinal$'max.heur.number'=="20"]

t.test(MH20Hamrug8tournrelaya, MH20Hamrug8tournrelayb, paired=T)

MH20Hamrug10tournrelaya <- HPDFinal$'tourn.of.relays.randoms'[HPDFinal$SmoothingFactor=="10" & HPDFinal$'max.heur.number'=="20"]

MH20Hamrug10tournrelayb <- HPDFinal$'tourn.of.relays.experts'[HPDFinal$SmoothingFactor=="10" & HPDFinal$'max.heur.number'=="20"]

t.test(MH20Hamrug10tournrelaya, MH20Hamrug10tournrelayb, paired=T)

MH20Hamrug12tournrelaya <- HPDFinal$'tourn.of.relays.randoms'[HPDFinal$SmoothingFactor=="12" & HPDFinal$'max.heur.number'=="20"]

MH20Hamrug12tournrelayb <- HPDFinal$'tourn.of.relays.experts'[HPDFinal$SmoothingFactor=="12" & HPDFinal$'max.heur.number'=="20"]

t.test(MH20Hamrug12tournrelaya, MH20Hamrug12tournrelayb, paired=T)

MH20Hamrug14tournrelaya <- HPDFinal$'tourn.of.relays.randoms'[HPDFinal$SmoothingFactor=="14" & HPDFinal$'max.heur.number'=="20"]

MH20Hamrug14tournrelayb <- HPDFinal$'tourn.of.relays.experts'[HPDFinal$SmoothingFactor=="14" & HPDFinal$'max.heur.number'=="20"]

t.test(MH20Hamrug14tournrelaya, MH20Hamrug14tournrelayb, paired=T)

MH20Hamrug16tournrelaya <- HPDFinal$'tourn.of.relays.randoms'[HPDFinal$SmoothingFactor=="16" & HPDFinal$'max.heur.number'=="20"]

MH20Hamrug16tournrelayb <- HPDFinal$'tourn.of.relays.experts'[HPDFinal$SmoothingFactor=="16" & HPDFinal$'max.heur.number'=="20"]

t.test(MH20Hamrug16tournrelaya, MH20Hamrug16tournrelayb, paired=T)

***

MH20Hamrug4Normtournrelaya <- HPDFinal$'tourn.of.relays.randoms.miscommunication'[HPDFinal$SmoothingFactor=="4" & HPDFinal$'max.heur.number'=="20" & HPDFinal$'distance.type'=="hamming" & HPDFinal$'distribution'=="normal" ]

MH20Hamrug4Normtournrelayb <- HPDFinal$'tourn.of.relays.experts.miscommunication'[HPDFinal$SmoothingFactor=="4" & HPDFinal$'max.heur.number'=="20" & HPDFinal$'distance.type'=="hamming" & HPDFinal$'distribution'=="normal"]

t.test(MH20Hamrug4Normtournrelaya, MH20Hamrug4Normtournrelayb, paired=T)

MH20Hamrug6Normtournrelaya <- HPDFinal$'tourn.of.relays.randoms.miscommunication'[HPDFinal$SmoothingFactor=="6" & HPDFinal$'max.heur.number'=="20" & HPDFinal$'distance.type'=="hamming" & HPDFinal$'distribution'=="normal" ]

MH20Hamrug6Normtournrelayb <- HPDFinal$'tourn.of.relays.experts.miscommunication'[HPDFinal$SmoothingFactor=="6" & HPDFinal$'max.heur.number'=="20" & HPDFinal$'distance.type'=="hamming" & HPDFinal$'distribution'=="normal"]

t.test(MH20Hamrug6Normtournrelaya, MH20Hamrug6Normtournrelayb, paired=T)

MH20Hamrug8Normtournrelaya <- HPDFinal$'tourn.of.relays.randoms.miscommunication'[HPDFinal$SmoothingFactor=="8" & HPDFinal$'max.heur.number'=="20" & HPDFinal$'distance.type'=="hamming" & HPDFinal$'distribution'=="normal" ]

MH20Hamrug8Normtournrelayb <- HPDFinal$'tourn.of.relays.experts.miscommunication'[HPDFinal$SmoothingFactor=="8" & HPDFinal$'max.heur.number'=="20" & HPDFinal$'distance.type'=="hamming" & HPDFinal$'distribution'=="normal"]

t.test(MH20Hamrug8Normtournrelaya, MH20Hamrug8Normtournrelayb, paired=T)

MH20Hamrug10Normtournrelaya <- HPDFinal$'tourn.of.relays.randoms.miscommunication'[HPDFinal$SmoothingFactor=="10" & HPDFinal$'max.heur.number'=="20" & HPDFinal$'distance.type'=="hamming" & HPDFinal$'distribution'=="normal" ]

MH20Hamrug10Normtournrelayb <- HPDFinal$'tourn.of.relays.experts.miscommunication'[HPDFinal$SmoothingFactor=="10" & HPDFinal$'max.heur.number'=="20" & HPDFinal$'distance.type'=="hamming" & HPDFinal$'distribution'=="normal"]

t.test(MH20Hamrug10Normtournrelaya, MH20Hamrug10Normtournrelayb, paired=T)

MH20Hamrug12Normtournrelaya <- HPDFinal$'tourn.of.relays.randoms.miscommunication'[HPDFinal$SmoothingFactor=="12" & HPDFinal$'max.heur.number'=="20" & HPDFinal$'distance.type'=="hamming" & HPDFinal$'distribution'=="normal" ]

MH20Hamrug12Normtournrelayb <- HPDFinal$'tourn.of.relays.experts.miscommunication'[HPDFinal$SmoothingFactor=="12" & HPDFinal$'max.heur.number'=="20" & HPDFinal$'distance.type'=="hamming" & HPDFinal$'distribution'=="normal"]

t.test(MH20Hamrug12Normtournrelaya, MH20Hamrug12Normtournrelayb, paired=T)

MH20Hamrug14Normtournrelaya <- HPDFinal$'tourn.of.relays.randoms.miscommunication'[HPDFinal$SmoothingFactor=="14" & HPDFinal$'max.heur.number'=="20" & HPDFinal$'distance.type'=="hamming" & HPDFinal$'distribution'=="normal" ]

MH20Hamrug14Normtournrelayb <- HPDFinal$'tourn.of.relays.experts.miscommunication'[HPDFinal$SmoothingFactor=="14" & HPDFinal$'max.heur.number'=="20" & HPDFinal$'distance.type'=="hamming" & HPDFinal$'distribution'=="normal"]

t.test(MH20Hamrug14Normtournrelaya, MH20Hamrug14Normtournrelayb, paired=T)

MH20Hamrug16Normtournrelaya <- HPDFinal$'tourn.of.relays.randoms.miscommunication'[HPDFinal$SmoothingFactor=="16" & HPDFinal$'max.heur.number'=="20" & HPDFinal$'distance.type'=="hamming" & HPDFinal$'distribution'=="normal" ]

MH20Hamrug16Normtournrelayb <- HPDFinal$'tourn.of.relays.experts.miscommunication'[HPDFinal$SmoothingFactor=="16" & HPDFinal$'max.heur.number'=="20" & HPDFinal$'distance.type'=="hamming" & HPDFinal$'distribution'=="normal"]

t.test(MH20Hamrug16Normtournrelaya, MH20Hamrug16Normtournrelayb, paired=T)

***

MH20Hamrug4Poistournrelaya <- HPDFinal$'tourn.of.relays.randoms.miscommunication'[HPDFinal$SmoothingFactor=="4" & HPDFinal$'max.heur.number'=="20" & HPDFinal$'distance.type'=="hamming" & HPDFinal$'distribution'=="poisson" ]

MH20Hamrug4Poistournrelayb <- HPDFinal$'tourn.of.relays.experts.miscommunication'[HPDFinal$SmoothingFactor=="4" & HPDFinal$'max.heur.number'=="20" & HPDFinal$'distance.type'=="hamming" & HPDFinal$'distribution'=="poisson"]

t.test(MH20Hamrug4Poistournrelaya, MH20Hamrug4Poistournrelayb, paired=T)

MH20Hamrug6Poistournrelaya <- HPDFinal$'tourn.of.relays.randoms.miscommunication'[HPDFinal$SmoothingFactor=="6" & HPDFinal$'max.heur.number'=="20" & HPDFinal$'distance.type'=="hamming" & HPDFinal$'distribution'=="poisson" ]

MH20Hamrug6Poistournrelayb <- HPDFinal$'tourn.of.relays.experts.miscommunication'[HPDFinal$SmoothingFactor=="6" & HPDFinal$'max.heur.number'=="20" & HPDFinal$'distance.type'=="hamming" & HPDFinal$'distribution'=="poisson"]

t.test(MH20Hamrug6Poistournrelaya, MH20Hamrug6Poistournrelayb, paired=T)

MH20Hamrug8Poistournrelaya <- HPDFinal$'tourn.of.relays.randoms.miscommunication'[HPDFinal$SmoothingFactor=="8" & HPDFinal$'max.heur.number'=="20" & HPDFinal$'distance.type'=="hamming" & HPDFinal$'distribution'=="poisson" ]

MH20Hamrug8Poistournrelayb <- HPDFinal$'tourn.of.relays.experts.miscommunication'[HPDFinal$SmoothingFactor=="8" & HPDFinal$'max.heur.number'=="20" & HPDFinal$'distance.type'=="hamming" & HPDFinal$'distribution'=="poisson"]

t.test(MH20Hamrug8Poistournrelaya, MH20Hamrug8Poistournrelayb, paired=T)

MH20Hamrug10Poistournrelaya <- HPDFinal$'tourn.of.relays.randoms.miscommunication'[HPDFinal$SmoothingFactor=="10" & HPDFinal$'max.heur.number'=="20" & HPDFinal$'distance.type'=="hamming" & HPDFinal$'distribution'=="poisson" ]

MH20Hamrug10Poistournrelayb <- HPDFinal$'tourn.of.relays.experts.miscommunication'[HPDFinal$SmoothingFactor=="10" & HPDFinal$'max.heur.number'=="20" & HPDFinal$'distance.type'=="hamming" & HPDFinal$'distribution'=="poisson"]

t.test(MH20Hamrug10Poistournrelaya, MH20Hamrug10Poistournrelayb, paired=T)

MH20Hamrug12Poistournrelaya <- HPDFinal$'tourn.of.relays.randoms.miscommunication'[HPDFinal$SmoothingFactor=="12" & HPDFinal$'max.heur.number'=="20" & HPDFinal$'distance.type'=="hamming" & HPDFinal$'distribution'=="poisson" ]

MH20Hamrug12Poistournrelayb <- HPDFinal$'tourn.of.relays.experts.miscommunication'[HPDFinal$SmoothingFactor=="12" & HPDFinal$'max.heur.number'=="20" & HPDFinal$'distance.type'=="hamming" & HPDFinal$'distribution'=="poisson"]

t.test(MH20Hamrug12Poistournrelaya, MH20Hamrug12Poistournrelayb, paired=T)

MH20Hamrug14Poistournrelaya <- HPDFinal$'tourn.of.relays.randoms.miscommunication'[HPDFinal$SmoothingFactor=="14" & HPDFinal$'max.heur.number'=="20" & HPDFinal$'distance.type'=="hamming" & HPDFinal$'distribution'=="poisson" ]

MH20Hamrug14Poistournrelayb <- HPDFinal$'tourn.of.relays.experts.miscommunication'[HPDFinal$SmoothingFactor=="14" & HPDFinal$'max.heur.number'=="20" & HPDFinal$'distance.type'=="hamming" & HPDFinal$'distribution'=="poisson"]

t.test(MH20Hamrug14Poistournrelaya, MH20Hamrug14Poistournrelayb, paired=T)

MH20Hamrug16Poistournrelaya <- HPDFinal$'tourn.of.relays.randoms.miscommunication'[HPDFinal$SmoothingFactor=="16" & HPDFinal$'max.heur.number'=="20" & HPDFinal$'distance.type'=="hamming" & HPDFinal$'distribution'=="poisson" ]

MH20Hamrug16Poistournrelayb <- HPDFinal$'tourn.of.relays.experts.miscommunication'[HPDFinal$SmoothingFactor=="16" & HPDFinal$'max.heur.number'=="20" & HPDFinal$'distance.type'=="hamming" & HPDFinal$'distribution'=="poisson"]

t.test(MH20Hamrug16Poistournrelaya, MH20Hamrug16Poistournrelayb, paired=T)

***

MH20Hamrug4Exptournrelaya <- HPDFinal$'tourn.of.relays.randoms.miscommunication'[HPDFinal$SmoothingFactor=="4" & HPDFinal$'max.heur.number'=="20" & HPDFinal$'distance.type'=="hamming" & HPDFinal$'distribution'=="exponential" ]

MH20Hamrug4Exptournrelayb <- HPDFinal$'tourn.of.relays.experts.miscommunication'[HPDFinal$SmoothingFactor=="4" & HPDFinal$'max.heur.number'=="20" & HPDFinal$'distance.type'=="hamming" & HPDFinal$'distribution'=="exponential"]

t.test(MH20Hamrug4Exptournrelaya, MH20Hamrug4Exptournrelayb, paired=T)

MH20Hamrug6Exptournrelaya <- HPDFinal$'tourn.of.relays.randoms.miscommunication'[HPDFinal$SmoothingFactor=="6" & HPDFinal$'max.heur.number'=="20" & HPDFinal$'distance.type'=="hamming" & HPDFinal$'distribution'=="exponential" ]

MH20Hamrug6Exptournrelayb <- HPDFinal$'tourn.of.relays.experts.miscommunication'[HPDFinal$SmoothingFactor=="6" & HPDFinal$'max.heur.number'=="20" & HPDFinal$'distance.type'=="hamming" & HPDFinal$'distribution'=="exponential"]

t.test(MH20Hamrug6Exptournrelaya, MH20Hamrug6Exptournrelayb, paired=T)

MH20Hamrug8Exptournrelaya <- HPDFinal$'tourn.of.relays.randoms.miscommunication'[HPDFinal$SmoothingFactor=="8" & HPDFinal$'max.heur.number'=="20" & HPDFinal$'distance.type'=="hamming" & HPDFinal$'distribution'=="exponential" ]

MH20Hamrug8Exptournrelayb <- HPDFinal$'tourn.of.relays.experts.miscommunication'[HPDFinal$SmoothingFactor=="8" & HPDFinal$'max.heur.number'=="20" & HPDFinal$'distance.type'=="hamming" & HPDFinal$'distribution'=="exponential"]

t.test(MH20Hamrug8Exptournrelaya, MH20Hamrug8Exptournrelayb, paired=T)

MH20Hamrug10Exptournrelaya <- HPDFinal$'tourn.of.relays.randoms.miscommunication'[HPDFinal$SmoothingFactor=="10" & HPDFinal$'max.heur.number'=="20" & HPDFinal$'distance.type'=="hamming" & HPDFinal$'distribution'=="exponential" ]

MH20Hamrug10Exptournrelayb <- HPDFinal$'tourn.of.relays.experts.miscommunication'[HPDFinal$SmoothingFactor=="10" & HPDFinal$'max.heur.number'=="20" & HPDFinal$'distance.type'=="hamming" & HPDFinal$'distribution'=="exponential"]

t.test(MH20Hamrug10Exptournrelaya, MH20Hamrug10Exptournrelayb, paired=T)

MH20Hamrug12Exptournrelaya <- HPDFinal$'tourn.of.relays.randoms.miscommunication'[HPDFinal$SmoothingFactor=="12" & HPDFinal$'max.heur.number'=="20" & HPDFinal$'distance.type'=="hamming" & HPDFinal$'distribution'=="exponential" ]

MH20Hamrug12Exptournrelayb <- HPDFinal$'tourn.of.relays.experts.miscommunication'[HPDFinal$SmoothingFactor=="12" & HPDFinal$'max.heur.number'=="20" & HPDFinal$'distance.type'=="hamming" & HPDFinal$'distribution'=="exponential"]

t.test(MH20Hamrug12Exptournrelaya, MH20Hamrug12Exptournrelayb, paired=T)

MH20Hamrug14Exptournrelaya <- HPDFinal$'tourn.of.relays.randoms.miscommunication'[HPDFinal$SmoothingFactor=="14" & HPDFinal$'max.heur.number'=="20" & HPDFinal$'distance.type'=="hamming" & HPDFinal$'distribution'=="exponential" ]

MH20Hamrug14Exptournrelayb <- HPDFinal$'tourn.of.relays.experts.miscommunication'[HPDFinal$SmoothingFactor=="14" & HPDFinal$'max.heur.number'=="20" & HPDFinal$'distance.type'=="hamming" & HPDFinal$'distribution'=="exponential"]

t.test(MH20Hamrug14Exptournrelaya, MH20Hamrug14Exptournrelayb, paired=T)

MH20Hamrug16Exptournrelaya <- HPDFinal$'tourn.of.relays.randoms.miscommunication'[HPDFinal$SmoothingFactor=="16" & HPDFinal$'max.heur.number'=="20" & HPDFinal$'distance.type'=="hamming" & HPDFinal$'distribution'=="exponential" ]

MH20Hamrug16Exptournrelayb <- HPDFinal$'tourn.of.relays.experts.miscommunication'[HPDFinal$SmoothingFactor=="16" & HPDFinal$'max.heur.number'=="20" & HPDFinal$'distance.type'=="hamming" & HPDFinal$'distribution'=="exponential"]

t.test(MH20Hamrug16Exptournrelaya, MH20Hamrug16Exptournrelayb, paired=T)

***

MH20Hamrug4Fixedtournrelaya <- HPDFinal$'tourn.of.relays.randoms.miscommunication.fixedsize'[HPDFinal$SmoothingFactor=="4" & HPDFinal$'max.heur.number'=="20" & HPDFinal$'distance.type'=="hamming" ]

MH20Hamrug4Fixedtournrelayb <- HPDFinal$'tourn.of.relays.experts.miscommunication.fixedsize'[HPDFinal$SmoothingFactor=="4" & HPDFinal$'max.heur.number'=="20" & HPDFinal$'distance.type'=="hamming" ]

t.test(MH20Hamrug4Fixedtournrelaya, MH20Hamrug4Fixedtournrelayb, paired=T)

MH20Hamrug6Fixedtournrelaya <- HPDFinal$'tourn.of.relays.randoms.miscommunication.fixedsize'[HPDFinal$SmoothingFactor=="6" & HPDFinal$'max.heur.number'=="20" & HPDFinal$'distance.type'=="hamming" ]

MH20Hamrug6Fixedtournrelayb <- HPDFinal$'tourn.of.relays.experts.miscommunication.fixedsize'[HPDFinal$SmoothingFactor=="6" & HPDFinal$'max.heur.number'=="20" & HPDFinal$'distance.type'=="hamming" ]

t.test(MH20Hamrug6Fixedtournrelaya, MH20Hamrug6Fixedtournrelayb, paired=T)

MH20Hamrug8Fixedtournrelaya <- HPDFinal$'tourn.of.relays.randoms.miscommunication.fixedsize'[HPDFinal$SmoothingFactor=="8" & HPDFinal$'max.heur.number'=="20" & HPDFinal$'distance.type'=="hamming" ]

MH20Hamrug8Fixedtournrelayb <- HPDFinal$'tourn.of.relays.experts.miscommunication.fixedsize'[HPDFinal$SmoothingFactor=="8" & HPDFinal$'max.heur.number'=="20" & HPDFinal$'distance.type'=="hamming" ]

t.test(MH20Hamrug8Fixedtournrelaya, MH20Hamrug8Fixedtournrelayb, paired=T)

MH20Hamrug10Fixedtournrelaya <- HPDFinal$'tourn.of.relays.randoms.miscommunication.fixedsize'[HPDFinal$SmoothingFactor=="10" & HPDFinal$'max.heur.number'=="20" & HPDFinal$'distance.type'=="hamming" ]

MH20Hamrug10Fixedtournrelayb <- HPDFinal$'tourn.of.relays.experts.miscommunication.fixedsize'[HPDFinal$SmoothingFactor=="10" & HPDFinal$'max.heur.number'=="20" & HPDFinal$'distance.type'=="hamming" ]

t.test(MH20Hamrug10Fixedtournrelaya, MH20Hamrug10Fixedtournrelayb, paired=T)

MH20Hamrug12Fixedtournrelaya <- HPDFinal$'tourn.of.relays.randoms.miscommunication.fixedsize'[HPDFinal$SmoothingFactor=="12" & HPDFinal$'max.heur.number'=="20" & HPDFinal$'distance.type'=="hamming" ]

MH20Hamrug12Fixedtournrelayb <- HPDFinal$'tourn.of.relays.experts.miscommunication.fixedsize'[HPDFinal$SmoothingFactor=="12" & HPDFinal$'max.heur.number'=="20" & HPDFinal$'distance.type'=="hamming" ]

t.test(MH20Hamrug12Fixedtournrelaya, MH20Hamrug12Fixedtournrelayb, paired=T)

MH20Hamrug14Fixedtournrelaya <- HPDFinal$'tourn.of.relays.randoms.miscommunication.fixedsize'[HPDFinal$SmoothingFactor=="14" & HPDFinal$'max.heur.number'=="20" & HPDFinal$'distance.type'=="hamming" ]

MH20Hamrug14Fixedtournrelayb <- HPDFinal$'tourn.of.relays.experts.miscommunication.fixedsize'[HPDFinal$SmoothingFactor=="14" & HPDFinal$'max.heur.number'=="20" & HPDFinal$'distance.type'=="hamming" ]

t.test(MH20Hamrug14Fixedtournrelaya, MH20Hamrug14Fixedtournrelayb, paired=T)

MH20Hamrug16Fixedtournrelaya <- HPDFinal$'tourn.of.relays.randoms.miscommunication.fixedsize'[HPDFinal$SmoothingFactor=="16" & HPDFinal$'max.heur.number'=="20" & HPDFinal$'distance.type'=="hamming" ]

MH20Hamrug16Fixedtournrelayb <- HPDFinal$'tourn.of.relays.experts.miscommunication.fixedsize'[HPDFinal$SmoothingFactor=="16" & HPDFinal$'max.heur.number'=="20" & HPDFinal$'distance.type'=="hamming" ]

t.test(MH20Hamrug16Fixedtournrelaya, MH20Hamrug16Fixedtournrelayb, paired=T)

**Hybrid Manhattan**

MH20Manrug4tournrelaya <- HPDFinal$'tourn.of.relays.randoms'[HPDFinal$SmoothingFactor=="4" & HPDFinal$'max.heur.number'=="20"]

MH20Manrug4tournrelayb <- HPDFinal$'tourn.of.relays.experts'[HPDFinal$SmoothingFactor=="4" & HPDFinal$'max.heur.number'=="20"]

t.test(MH20Manrug4tournrelaya, MH20Manrug4tournrelayb, paired=T)

MH20Manrug6tournrelaya <- HPDFinal$'tourn.of.relays.randoms'[HPDFinal$SmoothingFactor=="6" & HPDFinal$'max.heur.number'=="20"]

MH20Manrug6tournrelayb <- HPDFinal$'tourn.of.relays.experts'[HPDFinal$SmoothingFactor=="6" & HPDFinal$'max.heur.number'=="20"]

t.test(MH20Manrug6tournrelaya, MH20Manrug6tournrelayb, paired=T)

MH20Manrug8tournrelaya <- HPDFinal$'tourn.of.relays.randoms'[HPDFinal$SmoothingFactor=="8" & HPDFinal$'max.heur.number'=="20"]

MH20Manrug8tournrelayb <- HPDFinal$'tourn.of.relays.experts'[HPDFinal$SmoothingFactor=="8" & HPDFinal$'max.heur.number'=="20"]

t.test(MH20Manrug8tournrelaya, MH20Manrug8tournrelayb, paired=T)

MH20Manrug10tournrelaya <- HPDFinal$'tourn.of.relays.randoms'[HPDFinal$SmoothingFactor=="10" & HPDFinal$'max.heur.number'=="20"]

MH20Manrug10tournrelayb <- HPDFinal$'tourn.of.relays.experts'[HPDFinal$SmoothingFactor=="10" & HPDFinal$'max.heur.number'=="20"]

t.test(MH20Manrug10tournrelaya, MH20Manrug10tournrelayb, paired=T)

MH20Manrug12tournrelaya <- HPDFinal$'tourn.of.relays.randoms'[HPDFinal$SmoothingFactor=="12" & HPDFinal$'max.heur.number'=="20"]

MH20Manrug12tournrelayb <- HPDFinal$'tourn.of.relays.experts'[HPDFinal$SmoothingFactor=="12" & HPDFinal$'max.heur.number'=="20"]

t.test(MH20Manrug12tournrelaya, MH20Manrug12tournrelayb, paired=T)

MH20Manrug14tournrelaya <- HPDFinal$'tourn.of.relays.randoms'[HPDFinal$SmoothingFactor=="14" & HPDFinal$'max.heur.number'=="20"]

MH20Manrug14tournrelayb <- HPDFinal$'tourn.of.relays.experts'[HPDFinal$SmoothingFactor=="14" & HPDFinal$'max.heur.number'=="20"]

t.test(MH20Manrug14tournrelaya, MH20Manrug14tournrelayb, paired=T)

MH20Manrug16tournrelaya <- HPDFinal$'tourn.of.relays.randoms'[HPDFinal$SmoothingFactor=="16" & HPDFinal$'max.heur.number'=="20"]

MH20Manrug16tournrelayb <- HPDFinal$'tourn.of.relays.experts'[HPDFinal$SmoothingFactor=="16" & HPDFinal$'max.heur.number'=="20"]

t.test(MH20Manrug16tournrelaya, MH20Manrug16tournrelayb, paired=T)

***

MH20Manrug4Normtournrelaya <- HPDFinal$'tourn.of.relays.randoms.miscommunication'[HPDFinal$SmoothingFactor=="4" & HPDFinal$'max.heur.number'=="20" & HPDFinal$'distance.type'=="manhattan" & HPDFinal$'distribution'=="normal" ]

MH20Manrug4Normtournrelayb <- HPDFinal$'tourn.of.relays.experts.miscommunication'[HPDFinal$SmoothingFactor=="4" & HPDFinal$'max.heur.number'=="20" & HPDFinal$'distance.type'=="manhattan" & HPDFinal$'distribution'=="normal"]

t.test(MH20Manrug4Normtournrelaya, MH20Manrug4Normtournrelayb, paired=T)

MH20Manrug6Normtournrelaya <- HPDFinal$'tourn.of.relays.randoms.miscommunication'[HPDFinal$SmoothingFactor=="6" & HPDFinal$'max.heur.number'=="20" & HPDFinal$'distance.type'=="manhattan" & HPDFinal$'distribution'=="normal" ]

MH20Manrug6Normtournrelayb <- HPDFinal$'tourn.of.relays.experts.miscommunication'[HPDFinal$SmoothingFactor=="6" & HPDFinal$'max.heur.number'=="20" & HPDFinal$'distance.type'=="manhattan" & HPDFinal$'distribution'=="normal"]

t.test(MH20Manrug6Normtournrelaya, MH20Manrug6Normtournrelayb, paired=T)

MH20Manrug8Normtournrelaya <- HPDFinal$'tourn.of.relays.randoms.miscommunication'[HPDFinal$SmoothingFactor=="8" & HPDFinal$'max.heur.number'=="20" & HPDFinal$'distance.type'=="manhattan" & HPDFinal$'distribution'=="normal" ]

MH20Manrug8Normtournrelayb <- HPDFinal$'tourn.of.relays.experts.miscommunication'[HPDFinal$SmoothingFactor=="8" & HPDFinal$'max.heur.number'=="20" & HPDFinal$'distance.type'=="manhattan" & HPDFinal$'distribution'=="normal"]

t.test(MH20Manrug8Normtournrelaya, MH20Manrug8Normtournrelayb, paired=T)

MH20Manrug10Normtournrelaya <- HPDFinal$'tourn.of.relays.randoms.miscommunication'[HPDFinal$SmoothingFactor=="10" & HPDFinal$'max.heur.number'=="20" & HPDFinal$'distance.type'=="manhattan" & HPDFinal$'distribution'=="normal" ]

MH20Manrug10Normtournrelayb <- HPDFinal$'tourn.of.relays.experts.miscommunication'[HPDFinal$SmoothingFactor=="10" & HPDFinal$'max.heur.number'=="20" & HPDFinal$'distance.type'=="manhattan" & HPDFinal$'distribution'=="normal"]

t.test(MH20Manrug10Normtournrelaya, MH20Manrug10Normtournrelayb, paired=T)

MH20Manrug12Normtournrelaya <- HPDFinal$'tourn.of.relays.randoms.miscommunication'[HPDFinal$SmoothingFactor=="12" & HPDFinal$'max.heur.number'=="20" & HPDFinal$'distance.type'=="manhattan" & HPDFinal$'distribution'=="normal" ]

MH20Manrug12Normtournrelayb <- HPDFinal$'tourn.of.relays.experts.miscommunication'[HPDFinal$SmoothingFactor=="12" & HPDFinal$'max.heur.number'=="20" & HPDFinal$'distance.type'=="manhattan" & HPDFinal$'distribution'=="normal"]

t.test(MH20Manrug12Normtournrelaya, MH20Manrug12Normtournrelayb, paired=T)

MH20Manrug14Normtournrelaya <- HPDFinal$'tourn.of.relays.randoms.miscommunication'[HPDFinal$SmoothingFactor=="14" & HPDFinal$'max.heur.number'=="20" & HPDFinal$'distance.type'=="manhattan" & HPDFinal$'distribution'=="normal" ]

MH20Manrug14Normtournrelayb <- HPDFinal$'tourn.of.relays.experts.miscommunication'[HPDFinal$SmoothingFactor=="14" & HPDFinal$'max.heur.number'=="20" & HPDFinal$'distance.type'=="manhattan" & HPDFinal$'distribution'=="normal"]

t.test(MH20Manrug14Normtournrelaya, MH20Manrug14Normtournrelayb, paired=T)

MH20Manrug16Normtournrelaya <- HPDFinal$'tourn.of.relays.randoms.miscommunication'[HPDFinal$SmoothingFactor=="16" & HPDFinal$'max.heur.number'=="20" & HPDFinal$'distance.type'=="manhattan" & HPDFinal$'distribution'=="normal" ]

MH20Manrug16Normtournrelayb <- HPDFinal$'tourn.of.relays.experts.miscommunication'[HPDFinal$SmoothingFactor=="16" & HPDFinal$'max.heur.number'=="20" & HPDFinal$'distance.type'=="manhattan" & HPDFinal$'distribution'=="normal"]

t.test(MH20Manrug16Normtournrelaya, MH20Manrug16Normtournrelayb, paired=T)

***

MH20Manrug4Poistournrelaya <- HPDFinal$'tourn.of.relays.randoms.miscommunication'[HPDFinal$SmoothingFactor=="4" & HPDFinal$'max.heur.number'=="20" & HPDFinal$'distance.type'=="manhattan" & HPDFinal$'distribution'=="poisson" ]

MH20Manrug4Poistournrelayb <- HPDFinal$'tourn.of.relays.experts.miscommunication'[HPDFinal$SmoothingFactor=="4" & HPDFinal$'max.heur.number'=="20" & HPDFinal$'distance.type'=="manhattan" & HPDFinal$'distribution'=="poisson"]

t.test(MH20Manrug4Poistournrelaya, MH20Manrug4Poistournrelayb, paired=T)

MH20Manrug6Poistournrelaya <- HPDFinal$'tourn.of.relays.randoms.miscommunication'[HPDFinal$SmoothingFactor=="6" & HPDFinal$'max.heur.number'=="20" & HPDFinal$'distance.type'=="manhattan" & HPDFinal$'distribution'=="poisson" ]

MH20Manrug6Poistournrelayb <- HPDFinal$'tourn.of.relays.experts.miscommunication'[HPDFinal$SmoothingFactor=="6" & HPDFinal$'max.heur.number'=="20" & HPDFinal$'distance.type'=="manhattan" & HPDFinal$'distribution'=="poisson"]

t.test(MH20Manrug6Poistournrelaya, MH20Manrug6Poistournrelayb, paired=T)

MH20Manrug8Poistournrelaya <- HPDFinal$'tourn.of.relays.randoms.miscommunication'[HPDFinal$SmoothingFactor=="8" & HPDFinal$'max.heur.number'=="20" & HPDFinal$'distance.type'=="manhattan" & HPDFinal$'distribution'=="poisson" ]

MH20Manrug8Poistournrelayb <- HPDFinal$'tourn.of.relays.experts.miscommunication'[HPDFinal$SmoothingFactor=="8" & HPDFinal$'max.heur.number'=="20" & HPDFinal$'distance.type'=="manhattan" & HPDFinal$'distribution'=="poisson"]

t.test(MH20Manrug8Poistournrelaya, MH20Manrug8Poistournrelayb, paired=T)

MH20Manrug10Poistournrelaya <- HPDFinal$'tourn.of.relays.randoms.miscommunication'[HPDFinal$SmoothingFactor=="10" & HPDFinal$'max.heur.number'=="20" & HPDFinal$'distance.type'=="manhattan" & HPDFinal$'distribution'=="poisson" ]

MH20Manrug10Poistournrelayb <- HPDFinal$'tourn.of.relays.experts.miscommunication'[HPDFinal$SmoothingFactor=="10" & HPDFinal$'max.heur.number'=="20" & HPDFinal$'distance.type'=="manhattan" & HPDFinal$'distribution'=="poisson"]

t.test(MH20Manrug10Poistournrelaya, MH20Manrug10Poistournrelayb, paired=T)

MH20Manrug12Poistournrelaya <- HPDFinal$'tourn.of.relays.randoms.miscommunication'[HPDFinal$SmoothingFactor=="12" & HPDFinal$'max.heur.number'=="20" & HPDFinal$'distance.type'=="manhattan" & HPDFinal$'distribution'=="poisson" ]

MH20Manrug12Poistournrelayb <- HPDFinal$'tourn.of.relays.experts.miscommunication'[HPDFinal$SmoothingFactor=="12" & HPDFinal$'max.heur.number'=="20" & HPDFinal$'distance.type'=="manhattan" & HPDFinal$'distribution'=="poisson"]

t.test(MH20Manrug12Poistournrelaya, MH20Manrug12Poistournrelayb, paired=T)

MH20Manrug14Poistournrelaya <- HPDFinal$'tourn.of.relays.randoms.miscommunication'[HPDFinal$SmoothingFactor=="14" & HPDFinal$'max.heur.number'=="20" & HPDFinal$'distance.type'=="manhattan" & HPDFinal$'distribution'=="poisson" ]

MH20Manrug14Poistournrelayb <- HPDFinal$'tourn.of.relays.experts.miscommunication'[HPDFinal$SmoothingFactor=="14" & HPDFinal$'max.heur.number'=="20" & HPDFinal$'distance.type'=="manhattan" & HPDFinal$'distribution'=="poisson"]

t.test(MH20Manrug14Poistournrelaya, MH20Manrug14Poistournrelayb, paired=T)

MH20Manrug16Poistournrelaya <- HPDFinal$'tourn.of.relays.randoms.miscommunication'[HPDFinal$SmoothingFactor=="16" & HPDFinal$'max.heur.number'=="20" & HPDFinal$'distance.type'=="manhattan" & HPDFinal$'distribution'=="poisson" ]

MH20Manrug16Poistournrelayb <- HPDFinal$'tourn.of.relays.experts.miscommunication'[HPDFinal$SmoothingFactor=="16" & HPDFinal$'max.heur.number'=="20" & HPDFinal$'distance.type'=="manhattan" & HPDFinal$'distribution'=="poisson"]

t.test(MH20Manrug16Poistournrelaya, MH20Manrug16Poistournrelayb, paired=T)

***

MH20Manrug4Exptournrelaya <- HPDFinal$'tourn.of.relays.randoms.miscommunication'[HPDFinal$SmoothingFactor=="4" & HPDFinal$'max.heur.number'=="20" & HPDFinal$'distance.type'=="manhattan" & HPDFinal$'distribution'=="exponential" ]

MH20Manrug4Exptournrelayb <- HPDFinal$'tourn.of.relays.experts.miscommunication'[HPDFinal$SmoothingFactor=="4" & HPDFinal$'max.heur.number'=="20" & HPDFinal$'distance.type'=="manhattan" & HPDFinal$'distribution'=="exponential"]

t.test(MH20Manrug4Exptournrelaya, MH20Manrug4Exptournrelayb, paired=T)

MH20Manrug6Exptournrelaya <- HPDFinal$'tourn.of.relays.randoms.miscommunication'[HPDFinal$SmoothingFactor=="6" & HPDFinal$'max.heur.number'=="20" & HPDFinal$'distance.type'=="manhattan" & HPDFinal$'distribution'=="exponential" ]

MH20Manrug6Exptournrelayb <- HPDFinal$'tourn.of.relays.experts.miscommunication'[HPDFinal$SmoothingFactor=="6" & HPDFinal$'max.heur.number'=="20" & HPDFinal$'distance.type'=="manhattan" & HPDFinal$'distribution'=="exponential"]

t.test(MH20Manrug6Exptournrelaya, MH20Manrug6Exptournrelayb, paired=T)

MH20Manrug8Exptournrelaya <- HPDFinal$'tourn.of.relays.randoms.miscommunication'[HPDFinal$SmoothingFactor=="8" & HPDFinal$'max.heur.number'=="20" & HPDFinal$'distance.type'=="manhattan" & HPDFinal$'distribution'=="exponential" ]

MH20Manrug8Exptournrelayb <- HPDFinal$'tourn.of.relays.experts.miscommunication'[HPDFinal$SmoothingFactor=="8" & HPDFinal$'max.heur.number'=="20" & HPDFinal$'distance.type'=="manhattan" & HPDFinal$'distribution'=="exponential"]

t.test(MH20Manrug8Exptournrelaya, MH20Manrug8Exptournrelayb, paired=T)

MH20Manrug10Exptournrelaya <- HPDFinal$'tourn.of.relays.randoms.miscommunication'[HPDFinal$SmoothingFactor=="10" & HPDFinal$'max.heur.number'=="20" & HPDFinal$'distance.type'=="manhattan" & HPDFinal$'distribution'=="exponential" ]

MH20Manrug10Exptournrelayb <- HPDFinal$'tourn.of.relays.experts.miscommunication'[HPDFinal$SmoothingFactor=="10" & HPDFinal$'max.heur.number'=="20" & HPDFinal$'distance.type'=="manhattan" & HPDFinal$'distribution'=="exponential"]

t.test(MH20Manrug10Exptournrelaya, MH20Manrug10Exptournrelayb, paired=T)

MH20Manrug12Exptournrelaya <- HPDFinal$'tourn.of.relays.randoms.miscommunication'[HPDFinal$SmoothingFactor=="12" & HPDFinal$'max.heur.number'=="20" & HPDFinal$'distance.type'=="manhattan" & HPDFinal$'distribution'=="exponential" ]

MH20Manrug12Exptournrelayb <- HPDFinal$'tourn.of.relays.experts.miscommunication'[HPDFinal$SmoothingFactor=="12" & HPDFinal$'max.heur.number'=="20" & HPDFinal$'distance.type'=="manhattan" & HPDFinal$'distribution'=="exponential"]

t.test(MH20Manrug12Exptournrelaya, MH20Manrug12Exptournrelayb, paired=T)

MH20Manrug14Exptournrelaya <- HPDFinal$'tourn.of.relays.randoms.miscommunication'[HPDFinal$SmoothingFactor=="14" & HPDFinal$'max.heur.number'=="20" & HPDFinal$'distance.type'=="manhattan" & HPDFinal$'distribution'=="exponential" ]

MH20Manrug14Exptournrelayb <- HPDFinal$'tourn.of.relays.experts.miscommunication'[HPDFinal$SmoothingFactor=="14" & HPDFinal$'max.heur.number'=="20" & HPDFinal$'distance.type'=="manhattan" & HPDFinal$'distribution'=="exponential"]

t.test(MH20Manrug14Exptournrelaya, MH20Manrug14Exptournrelayb, paired=T)

MH20Manrug16Exptournrelaya <- HPDFinal$'tourn.of.relays.randoms.miscommunication'[HPDFinal$SmoothingFactor=="16" & HPDFinal$'max.heur.number'=="20" & HPDFinal$'distance.type'=="manhattan" & HPDFinal$'distribution'=="exponential" ]

MH20Manrug16Exptournrelayb <- HPDFinal$'tourn.of.relays.experts.miscommunication'[HPDFinal$SmoothingFactor=="16" & HPDFinal$'max.heur.number'=="20" & HPDFinal$'distance.type'=="manhattan" & HPDFinal$'distribution'=="exponential"]

t.test(MH20Manrug16Exptournrelaya, MH20Manrug16Exptournrelayb, paired=T)

***

MH20Manrug4Fixedtournrelaya <- HPDFinal$'tourn.of.relays.randoms.miscommunication.fixedsize'[HPDFinal$SmoothingFactor=="4" & HPDFinal$'max.heur.number'=="20" & HPDFinal$'distance.type'=="manhattan" ]

MH20Manrug4Fixedtournrelayb <- HPDFinal$'tourn.of.relays.experts.miscommunication.fixedsize'[HPDFinal$SmoothingFactor=="4" & HPDFinal$'max.heur.number'=="20" & HPDFinal$'distance.type'=="manhattan" ]

t.test(MH20Manrug4Fixedtournrelaya, MH20Manrug4Fixedtournrelayb, paired=T)

MH20Manrug6Fixedtournrelaya <- HPDFinal$'tourn.of.relays.randoms.miscommunication.fixedsize'[HPDFinal$SmoothingFactor=="6" & HPDFinal$'max.heur.number'=="20" & HPDFinal$'distance.type'=="manhattan" ]

MH20Manrug6Fixedtournrelayb <- HPDFinal$'tourn.of.relays.experts.miscommunication.fixedsize'[HPDFinal$SmoothingFactor=="6" & HPDFinal$'max.heur.number'=="20" & HPDFinal$'distance.type'=="manhattan" ]

t.test(MH20Manrug6Fixedtournrelaya, MH20Manrug6Fixedtournrelayb, paired=T)

MH20Manrug8Fixedtournrelaya <- HPDFinal$'tourn.of.relays.randoms.miscommunication.fixedsize'[HPDFinal$SmoothingFactor=="8" & HPDFinal$'max.heur.number'=="20" & HPDFinal$'distance.type'=="manhattan" ]

MH20Manrug8Fixedtournrelayb <- HPDFinal$'tourn.of.relays.experts.miscommunication.fixedsize'[HPDFinal$SmoothingFactor=="8" & HPDFinal$'max.heur.number'=="20" & HPDFinal$'distance.type'=="manhattan" ]

t.test(MH20Manrug8Fixedtournrelaya, MH20Manrug8Fixedtournrelayb, paired=T)

MH20Manrug10Fixedtournrelaya <- HPDFinal$'tourn.of.relays.randoms.miscommunication.fixedsize'[HPDFinal$SmoothingFactor=="10" & HPDFinal$'max.heur.number'=="20" & HPDFinal$'distance.type'=="manhattan" ]

MH20Manrug10Fixedtournrelayb <- HPDFinal$'tourn.of.relays.experts.miscommunication.fixedsize'[HPDFinal$SmoothingFactor=="10" & HPDFinal$'max.heur.number'=="20" & HPDFinal$'distance.type'=="manhattan" ]

t.test(MH20Manrug10Fixedtournrelaya, MH20Manrug10Fixedtournrelayb, paired=T)

MH20Manrug12Fixedtournrelaya <- HPDFinal$'tourn.of.relays.randoms.miscommunication.fixedsize'[HPDFinal$SmoothingFactor=="12" & HPDFinal$'max.heur.number'=="20" & HPDFinal$'distance.type'=="manhattan" ]

MH20Manrug12Fixedtournrelayb <- HPDFinal$'tourn.of.relays.experts.miscommunication.fixedsize'[HPDFinal$SmoothingFactor=="12" & HPDFinal$'max.heur.number'=="20" & HPDFinal$'distance.type'=="manhattan" ]

t.test(MH20Manrug12Fixedtournrelaya, MH20Manrug12Fixedtournrelayb, paired=T)

MH20Manrug14Fixedtournrelaya <- HPDFinal$'tourn.of.relays.randoms.miscommunication.fixedsize'[HPDFinal$SmoothingFactor=="14" & HPDFinal$'max.heur.number'=="20" & HPDFinal$'distance.type'=="manhattan" ]

MH20Manrug14Fixedtournrelayb <- HPDFinal$'tourn.of.relays.experts.miscommunication.fixedsize'[HPDFinal$SmoothingFactor=="14" & HPDFinal$'max.heur.number'=="20" & HPDFinal$'distance.type'=="manhattan" ]

t.test(MH20Manrug14Fixedtournrelaya, MH20Manrug14Fixedtournrelayb, paired=T)

MH20Manrug16Fixedtournrelaya <- HPDFinal$'tourn.of.relays.randoms.miscommunication.fixedsize'[HPDFinal$SmoothingFactor=="16" & HPDFinal$'max.heur.number'=="20" & HPDFinal$'distance.type'=="manhattan" ]

MH20Manrug16Fixedtournrelayb <- HPDFinal$'tourn.of.relays.experts.miscommunication.fixedsize'[HPDFinal$SmoothingFactor=="16" & HPDFinal$'max.heur.number'=="20" & HPDFinal$'distance.type'=="manhattan" ]

t.test(MH20Manrug16Fixedtournrelaya, MH20Manrug16Fixedtournrelayb, paired=T)

**Dealing with the Data and Plotting Results**

**Clean R Code**

**HP_Data_Main <- read.csv("~/Downloads/HP_Data_Main.csv")**

**HPDMainClean <- HP_Data_Main[order(HP_Data_Main$X.run.number.),]**

**Relabeling Hamming -> Manhattan, and Match —> Hamming**

**HPDMainClean$distance.type <- as.character(HPDMainClean$distance.type)**

**> HPDFinal1 <- HPDMainClean**

**> HPDFinal1[HPDFinal1 == "hamming"] <-"manhattan"**

**> HPDFinal <- HPDFinal1**

**> HPDFinal[HPDFinal == "match"] <-"hamming"**

**> View(HPDFinal)**

**> HPDMainClean$distance.type <- as.factor(HPDMainClean$distance.type)**

**> HPDFinal1$distance.type <- as.factor(HPDFinal1$distance.type)**

**> HPDFinal$distance.type <- as.factor(HPDFinal$distance.type)**

**Export as Excel**

**install.packages("writexl")**

**library("writexl")**

**write_xlsx(HPDFinal,"Downloads\\HPDFinal.xlsx")**

**Producing a Simple Bar Plot**

**Step 0: Set up a dataframe**

**df <- data.frame(search=rep(c('relay', ‘tourn’), each=1), score=rep(c(.419, 1.17), each=1))**

***This is for Smoothing = 6, MH=20**

**Step 1: Make a Table with Desired Values**

**df1 <- df[-grep('search', colnames(df))] #eliminate first column specifying search type**

**dfMat <- data.matrix(df1) #dataframe to matrix**

**rownames(dfMat) <- c("relay", "tourn") #add in row names specifying search type**

**dfMat <- as.table(dfMat) #matrix to table**

**margin.table(dfMat,1) #yields a table with one row, two columns**

**Step 2: Use barplot() to produce the plot**

**tabl <- margin.table(dfMat,1)**

**barplot(tabl)**

**Producing a Less Simple Bar Plot I: MH=20, Sm=6, Communication=[perfect, fixed, normal]**

**Step 0: Set up a dataframe**

**df <- data.frame(search=rep(c('relay', 'tourn', 'relay-fixed', 'tourn-fixed', 'relay-normal', 'tourn-normal'), each=1), score=rep(c(.419, 1.17, -0.40, 0.78, 0.42, 1.26), each=1))**

***This is for Smoothing = 6, MH=20**

**Step 1: Make a Table with Desired Values**

**df1 <- df[-grep('search', colnames(df))] #eliminate first column specifying search type**

**dfMat <- data.matrix(df1) #dataframe to matrix**

**rownames(dfMat) <- c("relay", "tourn", "relay-fixedsize", "tourn-fixedsize", "relay-normal", "tourn-normal") #add in row names specifying search type**

**dfMat <- as.table(dfMat) #matrix to table**

**tabl <- margin.table(dfMat,1) #yields a table with one row, two columns**

**Step 2: Use barplot() to produce the plot**

**barplot(tabl)**

**Producing a Less Simple Bar Plot II: MH=20, Sm=6, Communication=[hybrid, Pois, exp]**

**Step 0: Set up a dataframe**

**dfx <- data.frame(search=rep(c('hybrid', 'relay-Pois', 'tourn-Poisson', 'relay-exponential', 'tourn-exponential'), each=1), score=rep(c(.87, 0.71, 0.53, 0.88, -2.10), each=1))**

***This is for Smoothing = 6, MH=20**

**Step 1: Make a Table with Desired Values**

**df1x <- dfx[-grep('search', colnames(dfx))] #eliminate first column specifying search type**

**dfMatx <- data.matrix(df1x) #dataframe to matrix**

**rownames(dfMatx) <- c("hybrid", "relay-Pois", "tourn-Poisson", "relay-exponential", "tourn-exponential") #add in row names specifying search type**

**dfMatx <- as.table(dfMatx) #matrix to table**

**tablx <- margin.table(dfMatx,1) #yields a table with one row, two columns**

**Step 2: Use barplot() to produce the plot**

**barplot(tablx)**

**Producing a Line Graph I: Manhattan Distance**

**Step 1: Define vectors s (smoothness) and performance scores, according to t-test results**

**s=c(4,6,8,10,12,14,16)**

**r=c(1.27,0.42,-0.19,-0.36,-0.42, -0.38,-0.36)**

**t=c(1.8,1.17,0.53,0.23,0.20,0.14,0.10)**

**m=c(-3.68,-6.20,-6.92,-5.39,-5.02,-3.98,-4.35)**

**n=c(0.67,*0.13*,-0.37,-0.56,-0.51,-0.54,-0.53)**

**o=c(1.96, 0.82, *0.01, -0.05, -0.12, -0.24, -0.15*) #italics are not Stat Significant at .05 level**

**p=c(1.50,0.84,0.25,*-0.14,-0.03,-0.13*,-0.21)**

**q=c(2.30,0.83,0.29,*-0.05,-0.22,-0.14,-0.09*)**

**u=c(0.85,0.39,-*0.03*,-0.31,-0.20,-0.23,-0.28)**

**v=c(3.00,1.08,*0.12,0.12,-0.18,0.04,-0.18*)**

**w=c(-1.83,-2.03,-2.08,-1.83,-1.62,-1.41,-1.17)**

**Step 2: Use plot() function to generate a plot, selecting colors and textures to represent search methods and diversity measures**

**-Blue solid = Relay with perfect communication (r)**

**plot(s,r, type="b", col="blue”, xlab=“Smoothness”, ylab=“Performance”, ylim=range(r,t))**

**-Blue dashed = Tournament with perfect communication (t)**

**lines(s,t, type="b", col="blue", lty = "dashed")**

**-Red solid= Relay with normal (o)**

**lines(s,o, type="b", col="red")**

**-Red dashed = Tourn with normal(p)**

**lines(s,p, type="b", col="red", lty = "dashed")**

**-Purple Solid = Relay Poisson (q)**

**lines(s,q, type="b", col=“purple”)**

**-Purple dashed = Tourn Poisson (u)**

**lines(s,u, type="b", col=“purple”, lty = "dashed")**

**-Orange Solid = Relay Exponential (v)**

**lines(s,v, type="b", col=“orange”)**

**-Orange dashed = Tourn Exponential (w)**

**lines(s,w, type="b", col=“orange”, lty = "dashed")**

**-Green solid = Relay with fixed miscomm (m)**

**lines(s,m, col=“green”)**

**-Green dashed = Tourn with fixed miscomm (n)**

**lines(s,n, col=“green”, lty = "dashed”)**

**Full Code**

**s=c(4,6,8,10,12,14,16)**

**r=c(1.27,0.42,-0.19,-0.36,-0.42, -0.38,-0.36)**

**t=c(1.8,1.17,0.53,0.23,0.20,0.14,0.10)**

**m=c(-3.68,-6.20,-6.92,-5.39,-5.02,-3.98,-4.35)**

**n=c(0.67,*0.13*,-0.37,-0.56,-0.51,-0.54,-0.53)**

**o=c(1.96, 0.82, *0.01, -0.05, -0.14, -0.24, -0.15*)**

**p=c(1.50,0.84,0.25,*-0.14,-0.03,-0.13*,-0.21)**

**q=c(2.30,0.83,0.29,*-0.05,-0.22,-0.14,-0.09*)**

**u=c(0.85,0.39,-*0.03*,-0.31,-0.20,-0.23,-0.28)**

**v=c(3.00,1.08,*0.12,0.12,-0.18,0.04,-0.18*)**

**w=c(-1.83,-2.03,-2.08,-1.83,-1.62,-1.41,-1.17)**

**plot(s,r, type="b", col="blue", xlab="Smoothness", ylab="Performance", ylim=range(r,t,m,n,o,q,u,v,w))**

**lines(s,t, type="b", col="blue", lty = "dashed")**

**lines(s,m, type="b", col="green")**

**lines(s,n, type="b", col="green", lty = "dashed")**

**lines(s,o, type="b", col="red")**

**lines(s,p, type="b", col="red", lty = "dashed")**

**lines(s,q, type="b", col="purple")**

**lines(s,u, type="b", col="purple", lty = "dashed")**

**lines(s,v, type="b", col="orange")**

**lines(s,w, type="b", col="orange", lty = "dashed")**

**title("Manhattan Distance")**

**legend(11,3.3,c("Perfect Communication","Normal", "Poisson","Exponential","Fixed"), lwd=c(4,4,4,4,4), col=c("blue", "red", "purple", "orange", "green"), y.intersp=1.1)**

**Producing a Line Graph II: Hamming (Match) Distance**

**Step 1: Define vectors s (smoothness) and performance scores, according to t-test results**

**s=c(4,6,8,10,12,14,16)**

**r=c(1.27,0.42,-0.19,-0.36,-0.42, -0.38,-0.36)**

**t=c(1.8,1.17,0.53,0.23,0.20,0.14,0.10) #italics are not Stat Significant at .05 level**

**c=c(1.34,0.42,-*0.14*,-0.30,-0.40,-0.25,-0.21)**

**d=c(1.87,1.26,0.40,0.23,*0.04,0.18*,0.21)**

**e=c(2.39,0.71,*0.10,0.10,0.05,-0.09,-0.05*)**

**f=c(1.21,0.53,-.53,-*0.08*,-0.20,-0.27,-0.31)**

**g=c(1.97,0.88,*0.14,0.13,-0.14,-0.07,-0.22*)**

**h=c(-1.87,-2.10,-1.83,-2.00,-1.53,-1.33,-1.26)**

**i=c(0.95,*-0.13*,-0.42,-0.74,-0.81,-0.55,-0.59)**

**j=c(1.38,0.78,0.19,*0.01,-0.08,-0.06,-0.11*)**

**Step 2: Use plot() function to generate a plot, selecting colors and textures to represent search methods and diversity measures**

**-Blue solid = Relay with perfect communication (r)**

**plot(s,r, type="b", col="blue”, xlab=“Smoothness”, ylab=“Performance”, ylim=range(r,t, c, d, e, f, g, h, i, j))**

**-Blue dashed = Tournament with perfect communication (t)**

**lines(s,t, type="b", col="blue", lty = "dashed")**

**-Red solid= Relay with normal (c)**

**lines(s,c, type="b", col="red")**

**-Red dashed = Tourn with normal(d)**

**lines(s,d, type="b", col="red", lty = "dashed")**

**-Purple Solid = Relay Poisson (e)**

**lines(s,e, type="b", col=“purple”)**

**-Purple dashed = Tourn Poisson (f)**

**lines(s,f, type="b", col="purple”, lty = "dashed")**

**-Orange Solid = Relay Exponential (g)**

**lines(s,g, type=“g”, col=“orange”)**

**-Orange dashed = Tourn Exponential (h)**

**lines(s,h, type=“h”, col=“orange”, lty = "dashed")**

**-Green solid = Relay with fixed miscomm (i)**

**lines(s,i, col=“green”)**

**-Green dashed = Tourn with fixed miscomm (j)**

**lines(s,j, col=“green”, lty = "dashed”)**

**-Yellow Solid = Hybrid**

**-Yellow dashed = Tourn fixed (y)**

**s=c(4,6,8,10,12,14,16)**

**r=c(1.27,0.42,-0.19,-0.36,-0.42, -0.38,-0.36)**

**t=c(1.8,1.17,0.53,0.23,0.20,0.14,0.10)**

**c=c(1.34,0.42,-0.14,-0.30,-0.40,-0.25,-0.21)**

**d=c(1.87,1.26,0.40,0.23,0.04,0.18,0.21)**

**e=c(2.39,0.71,0.10,0.10,0.05,-0.09,-0.05)**

**f=c(1.21,0.53,0.53,-0.08,-0.20,-0.27,-0.31)**

**g=c(1.97,0.88,0.14,0.13,-0.14,-0.07,-0.22)**

**h=c(-1.87,-2.10,-1.83,-2.00,-1.53,-1.33,-1.26)**

**i=c(0.95,-0.13,-0.42,-0.74,-0.81,-0.55,-0.59)**

**j=c(1.38,0.78,0.19,0.01,-0.08,-0.06,-0.11)**

**plot(s,r, type="b", col="blue", xlab="Smoothness", ylab="Performance", ylim=range(r,t, c, d, e, f, g, h, i, j))**

**lines(s,t, type="b", col="blue", lty = "dashed")**

**lines(s,c, type="b", col="red")**

**lines(s,d, type="b", col="red", lty = "dashed")**

**lines(s,e, type="b", col="purple")**

**lines(s,f, type="b", col="purple", lty = "dashed")**

**lines(s,g, type="b", col="orange")**

**lines(s,h, type="b", col="orange", lty = "dashed")**

**lines(s,i, type="b", col="green")**

**lines(s,j, type="b", col="green", lty = "dashed")**

**title("(a) Hamming Distance")**

**legend(11,2,c(“Perfect Communication","Normal", "Poisson","Exponential","Fixed"), lwd=c(4,4,4,4,4), col=c("blue", "red", "purple", "orange", "green"), y.intersp=1.1)**

**Producing a Line Graph III: Hybrid Search**

**Step 1: Define vectors s (smoothness) and performance scores, according to t-test results**

**s=c(4,6,8,10,12,14,16)**

**r=c(1.27,0.42,-0.19,-0.36,-0.42, -0.38,-0.36)**

**t=c(1.8,1.17,0.53,0.23,0.20,0.14,0.10)**

**hy=c(1.48,0.87,0.19-0.16,-0.16,-0.20,-0.11)**

**ah=c(*0.32,0.05*,-0.53,-0.52,-0.55,-0.46,-0.34) #italics are not Stat Significant at .05 level**

**bh=c(*0.03,*-0.66,-1.27,-1.06,-1.02,-0.95,-0.89)**

**ch=c(-0.28,-0.91,-1.32,-1.63,-1.26,-1.10,-0.97)**

**dh=c(0.22,*0.04*,-0.46,-0.57,-0.49,-0.49,-0.30)**

**am=c(*0.08*,-0.64,-1.02,-1.05,-0.76,-0.90,-0.67)**

**bm=c(-0.31,-0.81,-1.09,-1.29,-1.02,-0.99,-0.80)**

**cm=c(-*0.24,*-0.83,-1.18,-1.33,-1.31,-1.02,-0.94)**

**dm=c(-0.99,-1.74,-1.92,-1.93,-1.48,-1.29,-1.10)**

**Step 2: Use plot() function to generate a plot, selecting colors and textures to represent distance types and diversity measures**

**-Blue solid = Relay with perfect communication (r)**

**plot(s,r, type="b", col="blue”, xlab=“Smoothness”, ylab=“Performance”, ylim=range(r,t, ah,bh,ch,dh,am,bm,cm,dm))**

**-Blue dashed = Tournament with perfect communication (t)**

**lines(s,t, type="b", col="blue", lty = "dashed")**

**-Black Solid = Hybrid w perfect comm**

**-Red solid= Hamming Normal (ah)**

**lines(s,ah, type="b", col="red")**

**-Purple solid = Hamming Poisson (bh)**

**lines(s,bh, type="b", col=“purple”)**

**-Orange solid = Hamming Exponential (ch)**

**lines(s,ch, type="b", col=“orange”)**

**-Green solid = Hamming Fixed (dh)**

**lines(s,dh, type="b", col=“green”)**

**-Red dashed = Manhattan Normal (am)**

**lines(s,am, type=“b”, col=“red”, lty = "dashed")**

**-Purple dashed = Manhattan Poisson (bm)**

**lines(s,bm, type=“b”, col=“purple”, lty = "dashed")**

**-Orange dashed = Manhattan Exponential (cm)**

**lines(s,cm, type=“b”,col=“orange”, lty = "dashed”)**

**-Green dashed = Manhattan Fixed (dm)**

**lines(s,dm, type=“b”,col=“green”, lty = "dashed")**

**Full Code**

**HAMMING**

**s=c(4,6,8,10,12,14,16)**

**r=c(1.27,0.42,-0.19,-0.36,-0.42, -0.38,-0.36)**

**t=c(1.8,1.17,0.53,0.23,0.20,0.14,0.10)**

**hy=c(1.48,0.87,0.19,0.16,-0.16,-0.20,-0.11)**

**ah=c(0.32,0.05,-0.53,-0.52,-0.55,-0.46,-0.34)**

**bh=c(0.03,-0.66,-1.27,-1.06,-1.02,-0.95,-0.89)**

**ch=c(-0.28,-0.91,-1.32,-1.63,-1.26,-1.10,-0.97)**

**dh=c(0.22,0.04,-0.46,-0.57,-0.49,-0.49,-0.30)**

**am=c(0.08,-0.64,-1.02,-1.05,-0.76,-0.90,-0.67)**

**bm=c(-0.31,-0.81,-1.09,-1.29,-1.02,-0.99,-0.80)**

**cm=c(-0.24,-0.83,-1.18,-1.33,-1.31,-1.02,-0.94)**

**dm=c(-0.99,-1.74,-1.92,-1.93,-1.48,-1.29,-1.10)**

**plot(s,hy, type="b", col="blue", xlab="Smoothness", ylab="Performance", ylim=range(r,t,ah,bh,ch,dh,am,bm,cm,dm))**

**#lines(s,t, type="b", col="blue", lty = "dashed")**

**#lines(s,hy, type="b", col="black")**

**lines(s,ah, type="b", col="red")**

**lines(s,bh, type="b", col="purple")**

**lines(s,ch, type="b", col="orange")**

**lines(s,dh, type="b", col="green")**

**#lines(s,am, type="b", col="red", lty = "dashed")**

**#lines(s,bm, type="b", col="purple", lty = "dashed")**

**#lines(s,cm, type="b", col="orange", lty = "dashed")**

**#lines(s,dm, type="b",col="green", lty = "dashed")**

**title("(a) Hybrid Search with Hamming Distance")**

**legend(11,1.8,c("Perfect Communication","Normal", "Poisson","Exponential","Fixed"), lwd=c(4,4,4,4,4), col=c("blue”, "red", "purple", "orange", "green"), y.intersp=1.5)**

**MANHATTAN**

**plot(s,hy, type="b", col="blue", xlab="Smoothness", ylab="Performance", ylim=range(r,t,ah,bh,ch,dh,am,bm,cm,dm))**

**#lines(s,t, type="b", col="blue", lty = "dashed")**

**#lines(s,hy, type="b", col="black")**

**#lines(s,ah, type="b", col="red")**

**#lines(s,bh, type="b", col="purple")**

**#lines(s,ch, type="b", col="orange")**

**#lines(s,dh, type="b", col="green")**

**lines(s,am, type="b", col="red")**

**lines(s,bm, type="b", col="purple")**

**lines(s,cm, type="b", col="orange")**

**lines(s,dm, type="b",col="green")**

**title("(b) Hybrid Search with Manhattan Distance")**

**legend(11,1.8,c("Perfect Communication","Normal", "Poisson","Exponential","Fixed"), lwd=c(4,4,4,4,4), col=c("blue", "red", "purple", "orange", "green"), y.intersp=1.5)**

**Producing a Line Graph IV: Specialized, zoom-in plots**

**i) Hamming with normal, pois, and exp**

**plot(s,c, type="b", col="red", xlab="Smoothness", ylab="Performance", ylim=range(2.8,-.9))**

**#lines(s,t, type="b", col="blue", lty = "dashed")**

**lines(s,r, type="b", col="blue")**

**#lines(s,d, type="b", col="red", lty = "dashed")**

**lines(s,e, type="b", col="purple")**

**#lines(s,f, type="b", col="purple", lty = "dashed")**

**lines(s,g, type="b", col="orange")**

**#lines(s,h, type="b", col="orange", lty = "dashed")**

**#lines(s,i, type="b", col="green")**

**#lines(s,j, type="b", col="green", lty = "dashed")**

**title("(a) Hamming Distance")**

**legend(11,2,c("Perfect Communication", "Normal", "Poisson","Exponential"), lwd=c(4,4,4,4,4), col=c("blue","red", "purple", "orange"), y.intersp=1.1)**

**i) Manhattan with normal, pois, and exp**

**plot(s,r, type="b", col="blue", xlab="Smoothness", ylab="Performance", ylim=range(3,-.8))**

**#lines(s,m, type="b", col="green")**

**#lines(s,n, type="b", col="green", lty = "dashed")**

**lines(s,o, type="b", col="red")**

**#lines(s,p, type="b", col="red", lty = "dashed")**

**lines(s,q, type="b", col="purple")**

**#lines(s,u, type="b", col="purple", lty = "dashed")**

**lines(s,v, type="b", col="orange")**

**#lines(s,w, type="b", col="orange", lty = "dashed")**

**title("(b) Manhattan Distance")**

**legend(11,2.5,c("Perfect Communication","Normal", "Poisson","Exponential"), lwd=c(4,4,4,4,4), col=c("blue", "red", "purple", "orange"), y.intersp=1.1)**
