# Supplementary material for: Does (mis)communication mitigate the upshot of diversity?
Source: PLoS One. 2023 Mar 24;18(3):e0283248. doi: 10.1371/journal.pone.0283248 (PMC10038257; doi:10.1371/journal.pone.0283248)
Supplement: S1 Table — (PDF) [file pone.0283248.s004.pdf]

HP-Miscommunication Data Analysis Summary

Summary Table: MH4 Hamming (Match)

Smoothing	Relay	Tourn	Relay Norm	Tourn Norm	Relay Pois	Tourn Pois	Relay Exp	Tourn Exp	Relay Fixed	Tourn Fixed
4	-0.12	0.09	-0.02	0.07	.04	-.08	-.08	-.26	.46	-.07
6	-0.20	0.03	-0.16	-0.04	-.08	-.08	-.003	-.17	.26	-.05
10	-0.08	0.01	-0.08	-0.05	-.002	-.04	-.13	-.10	.23	-.03
12	-0.08	-.003	-0.05	-0.02	-.05	-.02	.07	-.07	.24	-.02

Summary Table: MH10 Hamming (Match)

Smoothing	Relay	Tourn	Relay Norm	Tourn Norm	Relay Pois	Tourn Pois	Relay Exp	Tourn Exp	Relay Fixed	Tourn Fixed
4	-0.18	0.18	-0.16	0.15	-0.04	-0.20	0.30	-1.80	-0.91	-0.04
6	-0.18	-0.04	-0.27	-0.04	-0.24	-0.33	-0.05	-1.28	-1.08	-0.19
10	-0.21	-0.01	-.09	-0.05	-0.08	-0.27	-0.02	-0.71	-0.48	-0.13
12	-0.13	-0.003	-0.17	-0.06	-0.18	-0.22	-0.002	-0.58	-0.27	-0.12

Summary Table: MH15 Hamming (Match)

Smoothing	Relay	Tourn	Relay Norm	Tourn Norm	Relay Pois	Tourn Pois	Relay Exp	Tourn Exp	Relay Fixed	Tourn Fixed
4	0.53	1.13	0.33	1.14	1.02	0.49	0.95	-1.98	-0.16	0.73
6	-0.13	0.42	-0.17	0.38	-0.04	-0.05	0.14	-1.69	-1.11	0.15
10	-0.31	0.07	-0.34	.003	-0.10	-0.25	.008	-1.41	-0.68	-0.12
12	-0.32	0.10	-0.22	0.04	0.16	-0.20	-0.04	-1.11	-0.53	-0.10

Summary Table: MH20 Hamming (Match)

Smoothin g	Relay	Tourn	Relay Norm	Tourn Norm	Relay Pois	Tourn Pois	Relay Exp	Tourn Exp	Relay Fixed	Tourn Fixed
4	1.27	1.8	1.34	1.87	2.39	1.21	1.97	-1.87	0.95	1.38

6	0.42	1.17	0.42	1.26	0.71	0.53	0.88	-2.10	-0.13	0.78
8	-0.19	0.53	-0.14	0.40	0.10	0.53	0.14	-1.83	-0.42	0.19
10	-0.36	0.23	-0.30	0.23	0.10	-0.08	0.13	-2.00	-0.74	0.01
12	-0.42	0.20	-0.40	0.04	0.05	-0.20	-0.14	-1.53	-0.81	-0.08
14	-0.38	0.14	-0.25	0.18	-0.09	-0.27	-0.07	-1.33	-0.55	-0.06
16	-0.36	0.10	-0.21	0.21	-0.05	-0.31	-0.22	-1.26	-0.59	-0.11

Summary Table: MH4 Manhattan

Smoothing	Relay	Tourn	Relay Normal	Tourn Normal	Relay Pois	Tourn Pois	Relay Exp	Tourn Exp	Relay Fixed	Tourn Fixed
4	-0.12	0.09	0.21	0.05	.05	-.09	-.12	-.22	-.10	-.06
6	-0.20	0.03	0.13	-0.12	-.02	-.09	-.001	-.17	.60	-.04
10	-0.08	0.01	0.09	-0.05	-.06	-.04	-.05	-.09	.44	-.02
12	-0.08	-.003	0.11	-0.07	-.08	-.03	.004	-.08	.24	-.01

Summary Table: MH10 Manhattan

Smoothing	Relay	Tourn	Relay Norm	Tourn Norm	Relay Pois	Tourn Pois	Relay Exp	Tourn Exp	Relay Fixed	Tourn Fixed
4	-0.18	0.18	0.37	0.09	-0.05	-0.10	0.005	-1.61	-5.08	-0.12
6	-0.18	-0.04	0.09	-0.19	0.12	-0.41	0.007	-1.54	-3.06	-0.43
10	-0.21	-0.01	0.13	-0.12	-0.05	-0.29	0.01	-0.79	-1.47	-0.30
12	-0.13	-0.003	0.21	-0.09	-0.11	-0.16	-0.13	-0.63	-0.98	-0.21

Summary Table: MH15 Manhattan

Smoothing	Relay	Tourn	Relay Norm	Tourn Norm	Relay Pois	Tourn Pois	Relay Exp	Tourn Exp	Relay Fixed	Tourn Fixed
4	0.53	1.13	0.95	0.87	.094	0.56	0.97	-1.89	-5.95	0.44
6	-0.13	0.42	0.03	0.24	0.14	-0.21	0.10	-1.86	-6.27	-0.23

10	-0.31	0.07	0.03	-0.09	-0.01	-0.29	-0.31	-1.46	-3.11	-0.46
12	-0.32	0.10	-0.08	-0.09	-0.09	-0.23	0..01	-1.13	-3.44	-0.39

Summary Table: MH20 Manhattan

Smoothing	Relay	Tourn	Relay Norm	Tourn Norm	Relay Pois	Tourn Pois	Relay Exp	Tourn Exp	Relay Fixed	Tourn Fixed
4	1.27	1.8	1.96	1.50	2.30	0.85	3.00	-1.83	-3.68	0.67
6	0.42	1.17	.82	0.84	0.83	0.39	1.08	-2.03	-6.20	0.13
8	-0.19	0.53	.01	0.25	0.29	-0.03	0.12	-2.08	-6.92	-0.37
10	-0.36	0.23	-0.05	-0.14	-0.05	-0.31	0.12	-1.83	-5.39	-0.56
12	-0.42	0.20	-0.14	-0.03	-0.22	-0.20	-0.18	-1.62	-5.02	-0.51
14	-0.38	0.14	-0.24	-0.13	-0.14	-0.23	0.04	-1.41	-3.98	-0.54
16	-0.36	0.10	-0.15	-0.21	-0.09	-0.28	-0.18	-1.17	-4.35	-0.53

Summary Table: MH20 Hamming (Match) Hybrid

Smoothing	Relay	Tourn	Hybrid	Hybrid Norm	Hybrid Pois	Hybrid Exp	Hybrid Fixed
4	1.27	1.8	1.48	0.32	0.03	-0.28	0.22
6	0.42	1.17	0.87	0.05	-0.66	-0.91	0.04
8	-0.19	0.53	0.19	-0.53	-1.27	-1.32	-0.46
10	-0.36	0.23	-0.16	-0.52	-1.06	-1.63	-0.57
12	-0.42	0.20	-0.16	-0.55	-1.02	-1.26	-0.49
14	-0.38	0.14	-0.20	-0.46	-0.95	-1.10	-0.49
16	-0.36	0.10	-0.11	-0.34	-0.89	-0.97	-0.30

Summary Table: MH20 Manhattan Hybrid

Smoothing	Relay	Tourn	Hybrid	Hybrid Norm	Hybrid Pois	Hybrid Exp	Hybrid Fixed

4	1.27	1.8	1.48	0.08	-0.31	-0.24	-0.99
6	0.42	1.17	0.87	-0.64	-0.81	-0.83	-1.74
8	-0.19	0.53	0.19	-1.02	-1.09	-1.18	-1.92
10	-0.36	0.23	-0.16	-1.05	-1.29	-1.33	-1.93
12	-0.42	0.20	-0.16	-0.76	-1.02	-1.31	-1.48
14	-0.38	0.14	-0.20	-0.90	-0.99	-1.02	-1.29
16	-0.36	0.10	-0.11	-0.67	-0.80	-0.94	-1.10
