# Supplementary material for: Does (mis)communication mitigate the upshot of diversity?
Source: PLoS One. 2023 Mar 24;18(3):e0283248. doi: 10.1371/journal.pone.0283248 (PMC10038257; doi:10.1371/journal.pone.0283248)
Supplement: Attachment — Submitted filename: Referee_report_on_PONE_D_22_19336.pdf [file pone.0283248.s005.pdf]

Referee report on the manuscript "Does (mis)communication mitigate the upshot of diversity?

submitted to: PLOS ONE (PONE-D-22-19336)

July 26, 2022

The goal of the paper is to include communication between agents into a model of problem solving by teams of diverse agents, whereby communication might occur with error, and the error might be correlated with agent diversity. The authors clarify the moderating roles of task complexity, the number of problem solving approaches, the ways of conceptualizing a problem, and institutional characteristics (in terms of search processes). I think the paper covers an interesting and important topic and is of interest to the diverse PLOS One readership. However, I also think that the authors should clarify some issues (in particular related to the model). I will be happy to read the rebuttals to my comments and reconsider my view on the manuscript.

Major concerns

1. On page 4, the authors relate the DTA theorem to the class of NK models, and introduce the fundamentals of the NK framework. On page 9, the describe that they extend the Hong-Page model. How is the NK framework related to the Hong-Page model? I think that the PLOS One readership with not be familiar with these models, and therefore, the authors should carefully discuss and compare the model (in particular since the DTA theorem is proven for the NK framework). What are the characteristics of landscapes generated using the two frameworks? In which respects to they differ?

2. I think that the model description provided in the paper is not very structures, and in consequence, it is very hard to follow. I highly recommend the authors to consult structured frameworks to introduce agent-based models (such as the ODD or the ODD+D protocol). Even if the authors decide not to follow these frameworks, they might get an idea of how to properly structure a model description and what information should be provided. For example,

(a) it is not clear what exactly is the agents' task. Do they explore the entire landscape and share information about the observed topology? To the jointly perform a task, divide the landscape into interdependent sub-landscapes (or sub-tasks) and the solution implemented in one time step is a a function of all agents' actions? In the first case, how sensitive are the results to the number of agents, i.e., can the global optimum be achieved more often and/or faster when more agents operate on the landscape?

(b) in Sec. 2.1, the authors discuss different approaches to measure diversity/similarity. How diversity/similarity is implemented, is not introduced. Usually, descriptions of agent-based models include descriptions of agents and their characteristics. It would perhaps be an option to introduce agents and their characteristics first, and then, second, discuss how to measure diversity/similarity. For example, as far as I understand, there are some implicit assumptions included in the model: (i) agents are diverse in their search strategies only, (ii) agents have the cognitive capacities to explore the entire landscape, (iii) it does not make a difference whether agents move 1 or 20 positions to explore the landscape (in terms of costs or effort), etc. I think that it is the authors job to make all assumptions explicit.

(c) In Sec. 2.2, the authors introduce their concepts of miscommunication. I think that it is required to properly introduce a model of information exchange first. How is information conceptualized? In the NK framework, locations of peaks are indicated by bitstrings. How is this piece of information implemented in the model introduced in this paper? Is it an index between 1 and 20,000? Again, as mentioned before, I suggest the authors to carefully compare the NK framework and their model.

(d) In Sec. 2.3, the authors introduce three modes of coordination. There is a lot of research on coordination in teams (that also employs the NK framework). This body of research analyzes the efficiency of coordination mechanisms in different settings (e.g., different task complexity, different patterns of interactions). How do the modes considered in this paper related to this body of research? For example, it is well known that sequential problem solving methods are particularly suited when the interactions (that shape the problem's complexity) follow a hierarchical pattern). How is this finding reflected in the results presented here?

(e) In Sec. 2.4, the authors introduce how their landscapes are generated. The section is called "Other parameters". Isn't it the case the landscapes are a crucial building block of the model? From a logical point of view, I would consider introducing the building blocks of the model in the following order: (i) landscapes on which agents operate, (ii) agents and their characteristics, (iii) structure of interactions between agents, (iv) modes of information exchange. Usually,

in agent-based models, agents are dynamic, i.e., they learn. How is learning included in this model? Are the agents' search strategies fixed during one simulation run or do the agents discover new search strategies?

(f) I think that the authors should consider adding a table that includes all model parameters and all values for these parameters considered in the simulation experiments.

(g) at the end of Sec. 2, the authors claim that the results are representative. Usually, I would expect a sensitivity analysis that proves this claim.

(h) How did the authors fix the number of simulation runs? What is the observation period (for how many time steps did the agents search on the landscape)? There are methods to fix the number of simulation runs and time steps described in the literature on simulation modelling, such as using the coefficient of variation to fix these values.

3. The authors discuss some related literature in Secs. 1.1 and 1.2. However, the discussion is very narrow in its focus. For example, a review of the effects of team diversity on group performance (that not explicitly mentions the DTA theorem) might allow to place the results in a wider context.

4. From a technical point of view, the authors present a model that includes different types of error that prevent groups of agents getting stuck at a local optimum (because errors cause some variability in the search process). This is an already established finding in different domains (and in particular in research that employs evolutionary optimization/hill climbing based search on rugged landscapes). I suggest the authors to include this aspect in the discussion since it describes the mechanism that drive most of the presented results. Also, I suggest the authors to be very precise with their contribution to the literature (given that this mechanism is already well known in different fields).

Minor concerns

1. On page 4, the authors introduce the fundamentals of the NK framework without explaining the main parameters N and K. It is well known in the domains of business and economics, that K (and also the patterns of interactions) play a crucial role in performance. The authors do not address this aspect.
