# Supplementary material for: Does (mis)communication mitigate the upshot of diversity?
Source: PLoS One. 2023 Mar 24;18(3):e0283248. doi: 10.1371/journal.pone.0283248 (PMC10038257; doi:10.1371/journal.pone.0283248)
Supplement: Attachment — Submitted filename: Reply to Referees.docx [file pone.0283248.s006.docx]

**Does (mis)communication mitigate the upshot of diversity?**

**Reply to Reviewers**

Thank you to Reviewers 1 and 2 for their close attention to the manuscript, and to the editor for giving us the chance to revise the manuscript. We extensively revised the manuscript in light of their comments. Doing so has substantially improved the clarity and rigor of our argument, and we hope that they agree that we’ve satisfactorily addressed the concerns they raised with the previous version. Our revisions to the manuscript focused on four things: 1) clarifying our discussion of the Hong-Page model of diversity and how it relates to the broader class of *NK* models, 2) utilizing the ODD protocol to clarify our presentation of the model, 3) describing how our model and the results we describe relate to other models on the relationship between diversity, communication, and team problem-solving, and 4) doing more to explain and justify our decision to exclude certain things from the model.

This document provides a detailed description of the revisions we made, as well a line by line response to particular comments and concerns raised by the reviewers. The next section provides a linear summary of the revisions we made to each section of the manuscript. Our response to Reviewer 1 follows that. And our response to Reviewer 2 concludes the document.

Revision Summary

Sec. 1.1

We extensively revised our discussion of the Hong-Page model of diversity in sec. 1.1. These revisions were focused on clarifying three things i) why we think the model is powerful and why we’re interested in extending it, ii) how to interpret the model, and iii) how the model relates to the broader class of *NK models*. The most significant revision here involved reorganizing the section to separate our discussion of the HP model and NK models. The discussion of the HP model is now presented first, with its relations to NK models only coming at the end. This avoids any confusion as to the similarities and differences between the model. We also clarified our presentation of the HP model itself, incorporated some of the results of a more extensive review of the literature and expanded upon our reasons for engaging with the model.

Sec. 1.2

We revised our discussion of communication frictions and diversity and our motivations for extending the HP model to account for other work that the reviewers drew our attention to. In particular we emphasize the relationship between our approach and the one adopted by Frigotto and Rossi (2012) that reviewer 2 helpfully drew our attention to. Like us Frigotto and Rossi engage with the Hong Page model, and again like us they are critical of its failure to incorporate communication. However, they are critical of other aspects of the HP model as well and so develop a competing modeling framework. We now discuss this issue at length and explain our decision to extend the HP framework as opposed to adopting the FR approach (roughly those are i) the HP model has been so widely cited that we want to adhere as closely to it as possible in order to better illustrate its limits, and ii) we think some of the worries registered by Frigotto and Rossi are overstated).

Sec. 2

The presentation of our model in section 2 was completely overhauled in order to conform to the ODD protocol that Reviewer 1 helpfully pointed us towards. This required us to reorganize our presentation of the model and expand upon or clarify our discussion of how certain components of the model work and how to interpret the model. In line with the ODD protocol this section now includes 7 subsections that more completely describe the model’s processes and components in a standard format. We also included a table in subsection 2.3 that summarizes the parameters included in the model and the values they take on.

Sec. 3 and 4

Revisions to the summary of results in section 3 and subsequent discussion in section 4 were both relatively minor and focused only on improving clarity. However, during the revision process we took the opportunity to review and analyze our data again, to satisfy ourselves that the results we present are a fair summary of our data and highlight the most important results. Reviewer 2 also noted that the quality of the figures was poor. The prior version we submitted rendered fine on our machines, but we’ve generated higher resolution versions of the figures that will hopefully render as higher quality images.

Sec. 5

Significant revisions were made to this section describing the limitations of our model and directions for future research. In particular, we used this section to highlight how our results fit into the broader literature and what new lines of inquiry seem most promising. Among the significant revisions here was further discussion of the Frigotto and Rossi 2012 paper that we also discussed in sec 1.2. Their work focuses on the intensity of communication where we focus on how agents communicate and what is lost in communication. As we argue, these approaches complement one another and point towards the possibility that future work could integrate these features. In addition to the Frigotto and Rossi paper we also highlight a paper by Fontinari and Rodrigues (2016) that explores the impact of connectivity in communication networks and landscape topology on collective problem-solving. The network structures they explore overlap in some ways with the team dynamics we explore, and the results they derive about the relationship between group size and connectivity point to promising lines of future research. Finally, following the advice of reviewer 2 we discuss the relationship between our model and the growing literature on social influence. Reviewer 2 is certainly right that a complete model of collective problem-solving in teams must account for social influence, and we further agree with their conjecture that diversity it plausibly a significant driver of why some agents are more influential than others. As we explain in the text, however, incorporating these dynamics into our model would likely make the results of the model significantly harder to interpret. In particular, while such a model might provide a more descriptively accurate picture of collective problem-solving it would not provide the same degree of critical leverage on the core question motivating this paper, namely whether the DTA result is robust to introducing a communication channel.

**Response to Reviewer 1**

We are grateful to Reviewer 1 for their constructive engagement with the manuscript, and especially for their very helpful suggestions for how the model might be more clearly presented.

Below are responses to the numbered comments they provided.

*1) NK Framework and the Hong-Page Model*

Reviewer 1 asked for greater clarity regarding the conceptual connection between the Hong-Page model of diversity and the NK complexity framework. To address this lack of clarity, we have reorganized section 1.1 where the Hong-Page model is introduced. We now introduce that model at greater length (**pp 4-6**) before turning to a discussion of how the model relates to the broader class of *NK* models (**p 7**).

The basic idea is that the complexity of the solution space can be understood in terms of the NK framework, while the ruggedness of the landscape upon which agents search in the Hong-Page model depends, in turn, on the complexity of the solution space. If there are many dimensions along which to optimize (high N), and if these dimensions are interdependent and non-linearly-related (i.e. non-additive), then the landscape will be extremely rugged. This occurs because small changes in the solution space will translate into huge changes in the quality of the resulting solution. As we now explain in the paper, we use the ruggedness of the landscape as a proxy for the complexity of the solutions, where complexity can be understood in terms of the NK framework.

In addition to providing explanation in the text, we have also cited several additional papers from the literature on collective problems solving that utilize NK models. These should give the reader a better sense of how our model fits into the broader literature, and should allow the curious reader to deepen their understanding of such models and how they’re deployed.

*2) Structure of our Description of the ABM*

Reviewer 1 pointed out that our presentation of the agent-based model (ABM) was a bit disorganized. To give it more organization, we have consulted the ODD protocol and used this protocol to restructure the presentation of the model in section 2.

On **pages 12-24**, we now walk through all seven elements of the ODD protocol. The new description is longer and more thorough, and it addresses the open questions raised by Reviewer 1 in points 2(a)-(c) and (e). In deciding to present things in this way, we have also taken note of Reviewer 1’s recommendation in comment 2(e) that we reorder the description of the model as follows: “(i) landscapes on which agents operate, (ii) agents and their characteristics, (iii) structure of interactions between agents, (iv) modes of information exchange.” The way in which the model is now presented following the order prescribed in the ODD protocol approximates reviewer 1’s suggestion. However there are some discrepancies owing to the ODD’s distinction between entities, state variables, and scales and processes. We follow the guidance of the OOD and now present our description of the landscape (i) and the agents that populate it (ii) in subsection 2.2 which details the state variable in the model, along with a description of the measures of diversity we utilize. Team search methods which correspond to (iii) and models of communication corresponding to (iv) are now introduced in subsection 2.3 which provides a process overview of the model, and are then explained in more detail in subsection 2.7 where the ODD protocol recommends providing complete description of the submodels implemented. We have also incorporated a table (**page 16**) that describes all of the parameters and possible parameter values, as Reviewer 1 suggested in 2(f).

In 2(d) reviewer 1 poses several questions about how the search methods we consider fit into the broader literature on coordination in teams. To address this issue we’ve done two things. First, in subsection 2.7 we’ve expanded on our discussion of the search methods we include in the model. The primary consideration guiding us here was to build a model that reflected the Hong-Page model as closely as possible. Second, we’ve also introduced a new discussion in section 5 that comments on the role search procedures play in driving our results and how these results fit into the broader literature. Particularly significant in this context is our discussion of the Fontanari and Rodrigues paper (**p 31**).

Finally, to address the points made in 2(g) and 2(h) we’ve expanded upon the discussion of data collection in subsection 2.4 and section 3. We appreciate the suggestion that providing a formal sensitivity analysis would bolster claims about the representativeness of our results. However, a review of the papers we cite reveals that this kind of analysis is rarely provided (at least in the papers we most closely engage with, e.g. Hong and Page (2004) or Frigotti and Rossi (2012)). For this this reason, and because the paper is already quite long, we’ve decided not to include such an analysis since our estimate was that it would not substantially contribute to how the results we report are interpreted.

*3) Related Literature*

Reviewer 1 suggested the literature we discussed in the prior manuscript was perhaps too narrow in its focus. In the course of revising the manuscript we conducted a much more extensive review of the literature and that is now reflected in several places, e.g:
- In subsection 1.1 we cite several papers that explore collective problem-solving in NK landscapes at **p 7**
- Our discussion of the reasons for incorporating miscommunication into the model in subsection 1.2 now cites several papers that explore similar phenomena, but that don’t engage with Hong and Page **pp 10-11**, and
- We include a more extensive discussion in section 5 of how our results fit into the broader literature and where there are promising lines for future inquiry **pp 30-31**.

4) *Technical Contribution*

Reviewer 1 suggests that “the authors present a model that includes different types of error that prevent groups of agents getting stuck at a local optimum (because errors cause some variability in the search process).” This is true and is an important part of the analysis. We’ve highlighted this more clearly than we did in the previous version of the manuscript (see e.g. lines 775-784 in sec. 2.7 and 920-930 in sec. 4). One thing to note, however, is that the result we identify here is not merely a reproduction of the established finding that introducing error or noise can prevent premature convergence on local optima. This is because the primary question we are investigating is how the introduction of this noise differentially impacts diverse and expert teams. The fact that introducing noise helps expert groups is thus part of the story, but only part of it.

**Response to Reviewer 2**

We are similarly grateful to reviewer 2 for their constructive engagement with the manuscript. In particular, their comments encouraged us to think more carefully about how our contribution fits into the broader literature, and they pointed us towards some papers that had escaped our attention in our initial survey of the literature. Below are responses to the numbered comments they provided.

1) *Frigotto and Rossi paper*

Reviewer 2 helpfully pointed us to a relevant study by Frigotto and Rossi. This paper was overlooked in our initial survey of the literature. We studied the model and found that Frigotto and Rossi focus on a different aspect of communication than we do. While they are primarily concerned with the level of communication, we are concerned with errors in communication. More significantly, where we extend the Hong-Page model they introduce a competing framework. We discuss these differences and our decision to extend the Hong-Page model (as opposed to introducing a competing model or extending the FR model) in subsection 1.2 at **p 10.** Roughly our judgment was that because the HP model has been so influential (and widely abused) it was rhetorically useful to build a model that kept as much of their framework as possible in order to better illustrate the limits of that modeling approach. That said, as we now suggest in section 5 at pp **30-31**, the question of how miscommunication of the sort we model interacts with the intensity of communication they model points toward a promising new research project.

It’s also worth noting that because the literature on collective problem-solving is exceptionally interdisciplinary reviewer 2’s suggestions that we look at the literature more closely were especially helpful not just because they drew our attention to a paper we had overlooked, but because they encouraged us to explore parts of the literature with which we were previously less familiar. Conducting this more thorough literature review allowed us to identify several other papers that have shaped how we think about where our paper fits in the literature and what directions the project might be taken going forward. Most of the fruits of this are described in our response to reviewer 1’s comment (3).

2) *Social influence*

Reviewer 2 notes that “As you add communication into the model, you should account for the fact that interacting agents not only share information but may also influence each other.” As we explained in our discussion of the revisions to section 5 of the manuscript Reviewer 2 is certainly right that a complete model of collective problem-solving in teams must account for social influence, and we further agree with their conjecture that diversity it plausibly a significant driver of why some agents are more influential than others. To account for this we have included a discussion of how our model and the results we derive from it relate to the growing literature on social influence (**p 31-32).** As we explain in the text, however, incorporating these dynamics into our model would likely make the results of the model significantly harder to interpret. In particular, while such a model might provide a more descriptively accurate picture of collective problem-solving it would not provide the same degree of critical leverage on the core question motivating this paper, namely whether the DTA result is robust to introducing a communication channel. For that reason we decided against revising our model along the lines reviewer 2 suggests. That said, as we say in the text of the manuscript, there are important questions to ask about how social influence is related to diversity and how accounting for this might complicate the lessons we drew here.

3) *Minor Comments*

We have addressed all of the smaller suggestions made by Reviewer 2:

- On **p 22**, we have added a simple example that explains how miscommunication manifests itself in the various search procedures.

- We have clarified the description of the ring-shaped landscape employed in the model in two places on **page 4**, where we describe the Hong-Page model, and in **section 2.2.1** where we describe how the landscape is generated in our mode. We’ve also made sure that our discussion of the landscape employed in our model and others like it is consistent throughout the paper. As we now explain more clearly, in the two-dimensional ring shaped landscape we consider one dimension corresponds to the similarity of solutions to a problem, this is arrayed on the x-axis and is the dimension along which agents move guided by their heuristics. The other dimension corresponds to the quality or “score” of the solutions and is depicted along the y-axis and often referred to as “height” in our discussion.

- We have corrected our confusing use of “section” where “subsection” would be more appropriate

- We have corrected several typos, including the one pointed out by Reviewer 2.

- The figures included in the original manuscript were made on MatLab. They rendered fine on our machine but we have submitted new higher resolution images that will hopefully render better.
